# Smart Image-Based Deep Learning System for Automated Quality Grading of Phalaenopsis Seedlings in Outsourced Production

**DOI:** 10.3390/s25247502

**Published:** 2025-12-10

**Authors:** Hong-Dar Lin, Zheng-Yuan Zhang, Chou-Hsien Lin

**Affiliations:** 1 Department of Industrial Engineering and Management, Chaoyang University of Technology, Taichung 413310, Taiwan; s11215603@cyut.edu.tw; 2 Department of Civil, Architectural and Environmental Engineering, The University of Texas at Austin, Austin, TX 78712, USA; chslin@utexas.edu

**Keywords:** Phalaenopsis quality grading, automated visual inspection, deep learning, YOLO model, SVM model, RGB-D images

## Abstract

Phalaenopsis orchids are one of Taiwan’s key floral export products, and maintaining consistent quality is crucial for international competitiveness. To improve production efficiency, many orchid farms outsource the early flask seedling stage to contract growers, who raise the plants to the 2.5-inch potted seedling stage before returning them for further greenhouse cultivation. Traditionally, the quality of these outsourced seedlings is evaluated manually by inspectors who visually detect defects and assign quality grades based on experience, a process that is time-consuming and subjective. This study introduces a smart image-based deep learning system for automatic quality grading of Phalaenopsis potted seedlings, combining computer vision, deep learning, and machine learning techniques to replace manual inspection. The system uses YOLOv8 and YOLOv10 models for defect and root detection, along with SVM and Random Forest classifiers for defect counting and grading. It employs a dual-view imaging approach, utilizing top-view RGB-D images to capture spatial leaf structures and multi-angle side-view RGB images to assess leaf and root conditions. Two grading strategies are developed: a three-stage hierarchical method that offers interpretable diagnostic results and a direct grading method for fast, end-to-end quality prediction. Performance comparisons and ablation studies show that using RGB-D top-view images and optimal viewing-angle combinations significantly improve grading accuracy. The system achieves F1-scores of 84.44% (three-stage) and 90.44% (direct), demonstrating high reliability and strong potential for automated quality assessment and export inspection in the orchid industry.

## 1. Introduction

Phalaenopsis orchids are among Taiwan’s most valuable export-oriented ornamental plants [1], with their commercial success heavily relying on consistent seedling quality during early production stages. In practice, quality inspection of outsourced 2.5-inch potted seedlings mainly depends on manual visual assessment, where inspectors evaluate defect types, root conditions, and overall appearance based on personal experience. However, this method suffers from inconsistency, subjectivity, and inefficiency, often causing grading disputes and fluctuating export quality. To address these issues, this study develops an automated, image-based quality grading system that performs defect detection, root grading, and overall quality classification. The aim is to replace manual inspection with a standardized, objective, and efficient grading process that improves reliability and scalability for orchid seedling production and export management.

**Figure 1:**
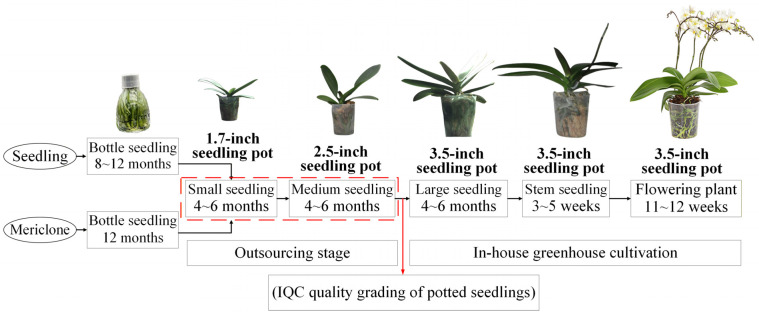
Phalaenopsis growth schedule and production methods in typical orchid gardens.

The cultivation process of Phalaenopsis orchids on a typical orchid farm can be divided into four production stages [2,3]. In the first stage, seedlings are produced from seeds or flower stalk nodes, mainly through meristem propagation, and grown into flask seedlings. In the second stage, the flask seedlings are outsourced to contractors for growing into potted seedlings of various sizes, starting with 1.7-inch pots. After 4–6 months, these seedlings are transplanted into 2.5-inch pots. In the third stage, the 2.5-inch seedlings raised by contractors undergo incoming quality control (IQC), where they are graded into three levels (A, B, and C), and outsourcing fees are paid based on grade. Once approved, the seedlings are further cultivated in-house for 4–6 months before being transplanted into 3.5-inch pots. In the fourth stage, outgoing quality control (OQC) is conducted according to customer requirements. After 4–6 months of growth, the 3.5-inch seedlings are shipped at different maturity stages, including large seedlings, spike-emerging seedlings, and flowering plants, with pricing determined by grade [3]. The growth timeline and production process of Phalaenopsis orchids on a typical orchid farm are shown in Figure 1.

**Figure 2:**
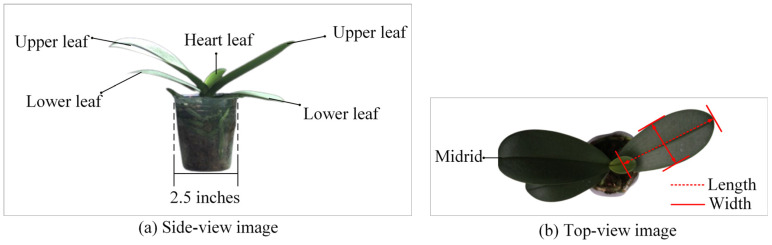
Leaf structure of Phalaenopsis seedlings in a 2.5-inch pot.

This study primarily focuses on outsourced 2.5-inch potted seedlings of large-flowered varieties, as illustrated in Figure 2. At this stage, the orchid typically has two upper leaves, two lower leaves, and one heart-shaped leaf, each with a midrib, as illustrated in Figure 2b. The central heart leaf is identified by observing the base of the leaf stem: each leaf has a growth termination line, but the heart leaf does not [4].

### 1.1. Defect Types in Phalaenopsis Potted Seedlings

During the seedling stage of Phalaenopsis orchids, the main types of defects include diseases, pest damage, phytotoxicity, leaf morphology issues such as leaf damage and leaf shrinkage, leaf surface problems like leaf variation and yellowing of lower leaves, and root system issues [3]. Each category can be further subdivided into specific items.

Common Phalaenopsis diseases include anthracnose, which begins as small discolored spots that merge into large concentric lesions turning from yellow-brown to dark brown; yellow leaf disease, a major export concern caused by desiccation and microbial growth during long transport, leading to leaf yellowing, thinning, wilting, and root rot; phytophthora disease, which starts as water-soaked lesions on mature leaves that expand into soft rot; southern blight (sclerotium disease), affecting roots and leaves with early water-soaked spots that develop white mycelium and later turn brown; bacterial soft rot, which causes water-soaked lesions on leaves, spikes, or petals that progress to drooping and decay; and brown spot disease, characterized by water-soaked spots that enlarge into necrotic streaks with yellow margins, eventually leading to leaf yellowing and abscission [5,6]. Examples of these six major diseases observed in Phalaenopsis seedlings are illustrated in Supplementary Figure S1.

During warm and dry seasons, Phalaenopsis seedlings are highly vulnerable to aphids, scale insects, and thrips [7,8]. Aphids hinder growth, deform leaves, spread viruses causing yellowing and poor flowering, and secrete sticky honeydew. Scale insects feed on plant sap, creating yellow or brown spots that cause leaf wilting and dropping. Thrips damage flowers and central leaves; flower buds shrink, turn yellow, or fall off; mature buds become wrinkled with white streaks; and central leaves twist and develop brown lesions. Examples of these three major insect damages in Phalaenopsis seedlings are shown in Supplementary Figure S2.

The three main types of phytotoxicity (pesticide damage) in Phalaenopsis orchids are caused by Vinclozolin, Dichlofluanid, and Metiram-vinclozolin [9]. Over 70% of affected plants exhibit yellowing or reddening at the leaf base, which may spread across the entire leaf or result in death in severe cases. These chemicals also shorten the flowering period, decrease flower production, and cause irregular inflorescences and lip deformities. Examples of these three common types of chemical damage in Phalaenopsis seedlings are shown in Supplementary Figure S3.

Leaf morphology disorders refer to visible damage on orchid seedling leaves, which, in severe cases, may lead to tearing. Leaf shrinkage, a common issue affecting the health and vigor of Phalaenopsis, is often caused by improper irrigation, nutrient deficiency, or environmental stress. It results in curled, yellowed, or browned leaves that eventually wither, greatly reducing flowering potential [10]. Examples of leaf damage and shrinkage in Phalaenopsis seedlings are illustrated in Supplementary Figure S4.

The surface of Phalaenopsis leaves can vary due to genetic traits, light exposure, or cultivation conditions. Common variations include variegation, twisted shapes, and slender leaves. Affected areas often show as raised, pale protrusions compared to normal tissue. Yellowing of lower leaves near the roots may result from nitrogen deficiency, excess moisture, or poor aeration, leading to leaf aging [10]. Examples of leaf variation and lower-leaf yellowing in Phalaenopsis seedlings are presented in Supplementary Figure S5.

Phalaenopsis orchids rely on healthy, vigorous roots for effective water and nutrient absorption, supporting robust growth, blooming, and prolonged flowering [11]. Root coverage over 70% of the pot is categorized as Grade A, 50–70% as Grade B, and less than 50% as Grade C. Examples of these three root conditions in Phalaenopsis seedlings are shown in Supplementary Figure S6.

### 1.2. Quality Grades for Acceptance of Outsourced Phalaenopsis Seedlings

**Figure 3:**
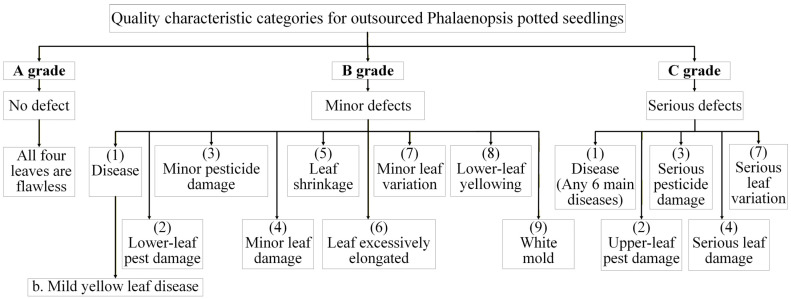
Quality characteristic categories for outsourced inspection and acceptance of Phalaenopsis potted seedlings.

**Figure 4:**
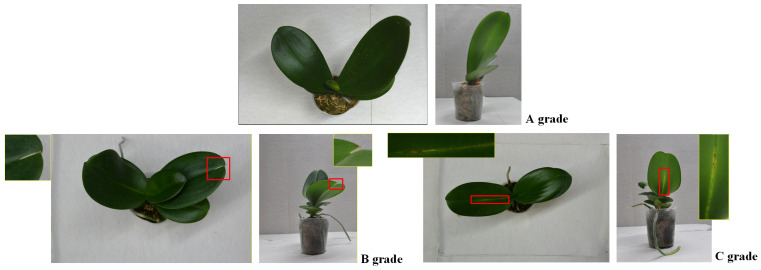
Images of seedlings meeting IQC Grades (**A**–**C**) (**left**: top-view image; **right**: side-view image), red squares indicating defect regions.

The quantitative grading indicators for IQC of large-flowered Phalaenopsis 2.5-inch seedlings are outlined as follows. Grade A seedlings must be nearly free of defects, showing no disease, pest damage, phytotoxicity, leaf injury, shrinkage, etiolation, variation, lower-leaf yellowing, or white mold, with healthy roots covering at least 70% of the pot. Grade B seedlings may have minor defects, such as slight disease, small bite marks, mild discoloration, or deformation, with damage less than 1.5 cm and root coverage between 50–70%. Grade C seedlings exhibit obvious defects, including disease spread, severe deformation, extensive yellowing, or leaf damage exceeding 1.5 cm, with poor roots covering less than 50% of the pot. Figure 3 illustrates inspection categories for outsourced Phalaenopsis seedlings; Table 1 provides details of the quantitative grading criteria used in IQC; and Figure 4 displays representative seedlings for Grades A–C.

### 1.3. Current Manual Quality Grading Processes

To ensure the quality of orchids during shipment, orchid farms employ quality control (QC) personnel to visually inspect Phalaenopsis at various growth stages. However, grading criteria often vary among inspectors, leading to inconsistent outcomes. Because QC staff rely solely on manual observation without measurement tools, the process is susceptible to subjective errors, visual fatigue, and decreased accuracy. Currently, farms outsource Phalaenopsis Sogo Yukidian ‘V3’ cultivation from the flask stage to the 2.5-inch pot stage, after which the seedlings are manually graded. Each inspection takes approximately 18 s (resulting in roughly 1600 seedlings per day), and contractor compensation depends on the assigned grade. Due to reliance on human judgment, misclassifications frequently occur, resulting in grading disputes that affect about 50% of seedlings [3].

### 1.4. Contribution

This study proposes a novel automated grading system for 2.5-inch outsourced Phalaenopsis seedlings, integrating multi-view imaging, depth sensing, and a hybrid deep learning–machine learning architecture. The main contributions are summarized as follows: (1) the development of a dual-modality (RGB-D + multi-view RGB) imaging pipeline tailored to 2.5-inch Phalaenopsis seedling inspection, (2) a hybrid multi-stage grading architecture integrating YOLO-based detection with SVM and RF decision models, (3) the introduction of an interpretable three-stage defect–root–quality reasoning framework, (4) a complementary direct grading model for rapid screening, and (5) extensive ablation and sensitivity analyses evaluating view-angle contributions, lighting robustness, and model-choice effects. Together, these contributions advance the state of the art in orchid seedling inspection and provide a practical, scalable foundation for automated quality control in commercial orchid production.

## 2. Literature Review

As demand for standardized export-quality production increases, integrating computer vision and deep learning offers a robust solution to the limitations of traditional manual inspection methods. This section reviews previous studies on Phalaenopsis production and grading practices, applications of machine vision in agriculture, recent advances in deep learning for defect detection, and hybrid learning frameworks for automated quality assessment.

### 2.1. Machine Vision Applications in Horticultural Quality Assessment

Machine vision techniques are widely used in horticulture for visual inspection tasks such as fruit sorting, disease detection, and flower grading [12,13,14]. Early methods relied on traditional image processing techniques, including color thresholding, edge detection, and texture analysis, to classify quality features or identify defects. While these approaches worked well under controlled conditions, they perform poorly in varying lighting, occlusion, or complex backgrounds [15]. The development of deep learning has largely overcome these limitations by enabling models to learn high-level, discriminative features directly from raw images.

In recent years, machine vision applications in horticulture have expanded beyond basic quality sorting to include complex tasks such as plant health monitoring, growth stage recognition, and phenotypic characterization. Studies have shown that combining spectral, morphological, and textural features enables a more comprehensive assessment of plant appearance and health status [16]. Furthermore, advances in imaging hardware, such as depth cameras and multispectral sensors, have improved the ability of vision systems to capture detailed three-dimensional and physiological information. These developments have shifted horticultural inspection from manual, subjective evaluation to automated, data-driven decision-making, providing a foundation for intelligent grading and defect detection systems, such as the one proposed in this study.

### 2.2. Deep Learning for Plant Defect Detection and Phenotyping

Deep convolutional neural networks (CNNs) have transformed agricultural image analysis, enabling reliable detection of plant diseases, pests, and morphological issues [17]. Object detection models, such as YOLO, Faster R-CNN, and EfficientDet, have achieved high accuracy in identifying small-scale symptoms and visual anomalies [18]. Recent variants, YOLOv8 and YOLOv10, provide faster real-time detection and better precision through more efficient network backbones and anchor-free architectures [19]. These features make them ideal for detecting various Phalaenopsis leaf defects, such as damage, yellowing, and deformation.

In addition to their strong detection capabilities, deep learning models have demonstrated significant potential in plant phenotyping, quantifying plant traits such as leaf count, shape, and size, which are critical indicators of plant vigor and growth quality [20]. By utilizing transfer learning and data augmentation, CNN-based models can generalize effectively across different species and environmental conditions, reducing the need for extensive labeled datasets [21]. Moreover, the combination of object detection and segmentation networks has enabled finer analysis of leaf morphology and stress symptoms, facilitating multi-trait phenotyping in complex canopy structures. These advancements highlight the adaptability of modern deep learning architectures for inspecting ornamental plants, making them an ideal foundation for developing an automated grading system for Phalaenopsis seedlings.

### 2.3. Integration of Depth Sensing (RGB-D) for Plant Morphological Analysis

RGB-D imaging combines traditional RGB data with depth maps to capture spatial structures and three-dimensional characteristics of plants [22]. In horticultural applications, RGB-D cameras such as Intel RealSense or Microsoft Kinect have been used for phenotyping tasks including canopy measurement, leaf segmentation, and growth tracking [23,24]. For Phalaenopsis seedlings, depth information is particularly valuable for distinguishing overlapping leaves and identifying shrinkage or deformation caused by environmental stress, improving both detection precision and interpretability of quality grading.

Beyond structural measurement, RGB-D imaging also enhances the robustness of plant analysis under variable lighting and complex backgrounds, conditions that often degrade the performance of conventional RGB-only systems [25]. The incorporation of depth data enables background removal, occlusion handling, and more accurate feature extraction of plant geometry, allowing for precise differentiation between healthy and defective regions. Recent studies have also demonstrated the effectiveness of fusing RGB-D data with deep learning networks, where depth channels serve as an additional modality that guides spatial attention and improves object segmentation accuracy [26]. Consequently, the integration of RGB-D imaging provides a strong foundation for developing a more reliable and interpretable system for detecting and grading defects in Phalaenopsis seedlings in greenhouse environments.

### 2.4. Multi-View and Multi-Modal Imaging Approaches in Plant Inspection

Because of the complex spatial geometry of plants, single-view imaging often fails to capture all relevant visual features. Multi-view imaging systems acquire data from multiple perspectives, typically using turntables or fixed-angle cameras, to provide more comprehensive visual coverage [27]. Combining top-view and side-view imagery enhances defect recognition and reduces the risk of missing occluded features. Multi-modal fusion, which integrates RGB, depth, and sometimes hyperspectral data, has been shown to improve classification robustness in crop disease detection and morphological trait analysis [28,29,30].

Multi-view and multi-modal imaging not only enhances visual completeness but also enables spatially aware feature learning, allowing models to understand the three-dimensional arrangement of plant organs and detect subtle defects that are invisible from a single angle [31]. When integrated with deep learning architectures, such systems can extract complementary information from each view—top views emphasizing leaf arrangement and color uniformity, while side views capture plant height, curvature, and root exposure. Recent research has shown that fusing multi-view data through attention-based or ensemble frameworks can significantly improve defect detection, maturity estimation, and quality grading accuracy [32,33]. These insights underscore the importance of multi-view integration in constructing a reliable, holistic inspection framework for Phalaenopsis seedlings.

### 2.5. Machine Learning for Feature-Based Classification and Grading

Classical machine learning algorithms, such as Support Vector Machines (SVMs) and Random Forests (RFs), have long been utilized for agricultural classification and grading tasks [34]. These models effectively handle structured features, including geometric measurements, color indices, and statistical summaries extracted from image data. When integrated with deep learning outputs, SVM and RF can perform secondary decision-making, such as quantifying defect severity or predicting overall quality categories [35]. This combination enables interpretable yet high-accuracy grading frameworks.

Beyond their predictive accuracy, machine learning models offer advantages in interpretability, flexibility, and computational efficiency, making them valuable for practical grading applications where transparency and speed are essential [36]. For instance, SVMs are particularly effective for small- to medium-sized datasets, offering robust boundary-based classification, while RFs excel in handling heterogeneous features and mitigating overfitting through ensemble averaging. Recent studies have also demonstrated that combining feature-level outputs from deep neural networks with classical classifiers enhances model generalization, especially when training data are limited or imbalanced [37,38]. Such hybrid frameworks bridge the gap between data-driven feature extraction and explainable decision-making, supporting the development of reliable and interpretable quality grading systems for Phalaenopsis seedlings.

### 2.6. Hybrid Deep Learning and Machine Learning Systems in Agriculture

Hybrid approaches that combine deep learning’s feature extraction capabilities with traditional machine learning classifiers have proven highly effective for tasks requiring interpretability and multi-stage reasoning [39]. Deep networks capture low- and mid-level image representations, while classical models refine decision boundaries based on engineered or aggregated features. Such methods have been successfully applied to fruit grading, plant disease diagnosis, and the classification of ornamental plants [40,41]. In this study, a similar hybrid design integrates YOLO-based detection models with SVM and Random Forest classifiers to achieve accurate, multi-stage quality grading of Phalaenopsis seedlings.

Hybrid deep learning and machine learning systems offer a balanced trade-off between automation, interpretability, and adaptability, making them particularly well-suited for agricultural inspection workflows where both accuracy and traceability are crucial [42]. While deep learning models excel in extracting complex spatial and textural patterns from raw imagery, machine learning classifiers offer transparent decision-making and easier parameter tuning for production-level deployment. Recent research has shown that such hybrid architectures outperform standalone deep learning systems when datasets are diverse or partially labeled, as the secondary classifiers can effectively integrate semantic, geometric, and statistical cues [43]. Therefore, combining deep feature extraction with feature-based classification provides a scalable pathway toward intelligent, interpretable, and industry-ready quality grading solutions for Phalaenopsis seedlings.

While numerous studies have explored plant disease detection and grading using image-based learning models, most focus on single-view or single-modality analysis. Few have addressed the integration of top-view RGB-D and multi-angle side-view imaging in a unified grading framework for ornamental crops. Furthermore, limited research has examined how hierarchical (multi-stage) and direct grading methods compare in terms of interpretability and efficiency. To bridge these gaps, this study develops a multi-view, multi-modal, hybrid deep learning and machine learning system that automates the quality grading of outsourced Phalaenopsis seedlings, enhancing accuracy, consistency, and industrial applicability. The proposed system introduces two grading strategies—a three-stage hierarchical method for interpretable diagnosis and a direct grading method for rapid assessment.

## 3. Materials and Methods

To address the limitations of manual inspection and enhance the consistency of seedling quality evaluation, this study proposes an automated grading framework for Phalaenopsis seedlings that integrates computer vision with deep learning and machine learning techniques. The proposed method follows a structured three-stage process: (I) detection of leaf-surface defects and estimation of root quantity using top-view RGB-D images and side-view RGB images, (II) transformation of detection results into quantitative features, including defect categories, defect counts, and root grading, and (III) integration of these features into a comprehensive seedling feature vector, which is subsequently classified using SVM and Random Forest models to determine final quality grades. This approach utilizes multi-view image acquisition and ensemble decision-making to improve accuracy, reduce subjectivity, and provide a standardized solution for large-scale seedling quality assessment.

**Figure 5:**
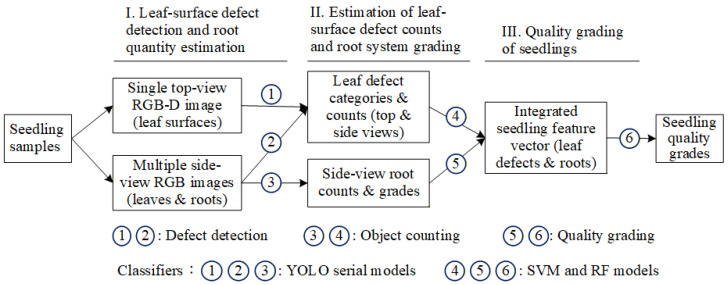
System concept diagram of the three-stage grading method in this study.

In this study, a depth camera and a digital camera are used to capture top-view RGB-D images and side-view multi-angle RGB images of Phalaenopsis seedlings. After image cropping and alignment correction, manual annotation is performed to establish labeled data for leaf surface defects and root quantity. The images and labeled data are then input into the YOLO model for defect detection and root estimation. The detection and estimation results are converted into feature vectors, which were subsequently fed into an SVM model for seedling quality grading. Finally, performance evaluations are conducted with other network models to select the optimal model combination. Figure 5 illustrates the system concept of the proposed three-stage grading method for Phalaenopsis seedlings. This pipeline ensures standardized, accurate, and efficient grading by combining multi-view imaging, deep learning, and machine learning approaches.

### 3.1. Image Acquisition of Potted Seedlings

**Figure 6:**
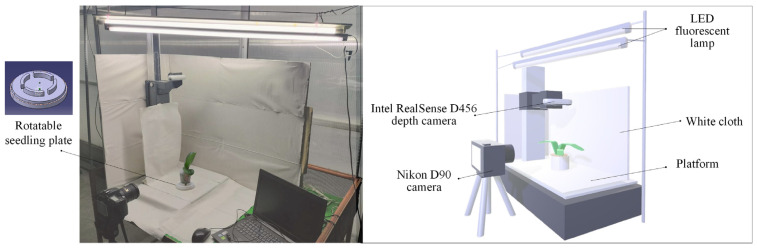
Hardware setup and schematic diagram of image acquisition in this study.

Accurate image acquisition is a critical foundation for automated defect detection and quality grading of Phalaenopsis seedlings. To obtain high-quality and consistent data, a controlled imaging setup is designed with multi-view capture capability, standardized lighting, and background conditions. This system enables the collection of both top-view RGB-D images and side-view RGB images, which are necessary for feature extraction and grading analysis. Figure 6 presents the hardware setup and schematic diagram used for image acquisition in this study. The experimental platform features a rotatable seedling plate that enables seedlings to be positioned at various viewing angles, facilitating multi-view image capture. Two cameras are employed: an Intel RealSense D456 depth camera for acquiring top-view RGB-D images and a Nikon D90 digital camera for capturing side-view RGB images of both leaves and roots. To ensure consistent illumination and minimize shadows, LED fluorescent lamps are installed above the platform, and white cloth is used as a background to provide a uniform contrast with the seedlings. This controlled setup enables the collection of high-quality images under standardized lighting and environmental conditions, forming the basis for subsequent defect detection and quality grading.

**Figure 7:**
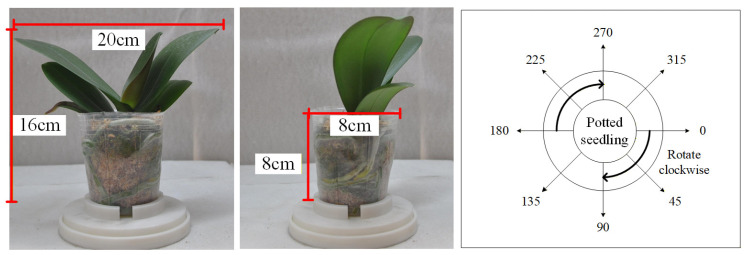
Practical images of the turntable at 0° and 90°, showing seedling dimensions and a schematic diagram of the rotation direction.

**Figure 8:**
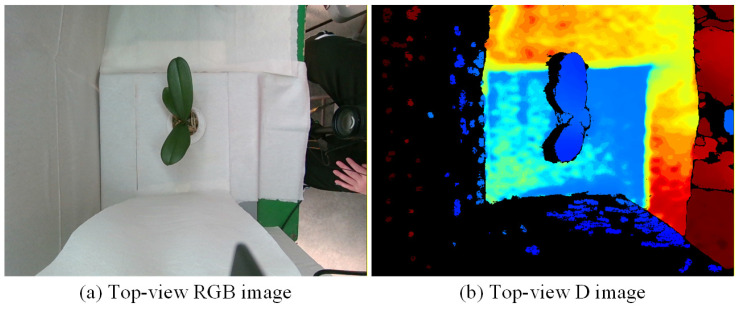
Captured top-view RGB and depth images of seedlings in this study.

**Figure 9:**
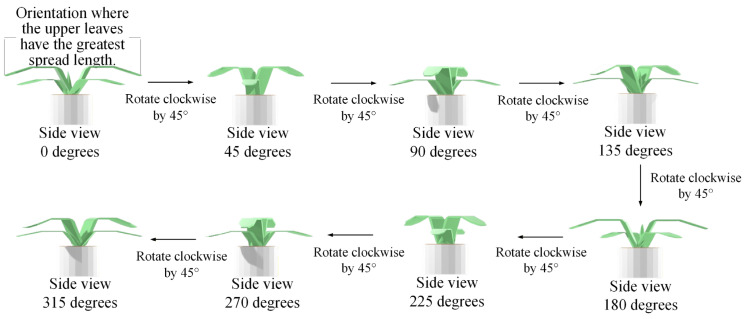
Schematic diagram showing the eight rotational angles used for side-view image acquisition in this study.

Figure 7, Figure 8 and Figure 9 illustrate the image acquisition process and multi-view setup used in this study. Figure 7 shows practical application images of the turntable at 0° and 90°, with seedling dimensions labeled, along with a schematic diagram indicating the clockwise rotation direction. Figure 8 presents examples of top-view images, including both an RGB image and a corresponding depth (D) image, which are used to capture color, texture, and structural features of seedlings. Figure 9 defines the eight side-view orientations used in this study, starting from 0° at the orientation where the upper leaves have the greatest spread and then rotating clockwise in 45° increments, ensuring comprehensive coverage of leaf and root structures, which, together with the top-view images, form the complete dataset for defect detection and quality grading. All eight side-view images were captured using a Nikon D90 camera at a resolution of 4288 × 2848 pixels. After acquisition, each image underwent fixed-offset cropping to remove peripheral background while maintaining full visibility of the seedling, resulting in a consistent processed resolution of 3038 × 2548 pixels for all side-view inputs.

### 3.2. Image Pre-Processing of Potted Seedlings

To enhance the accuracy and stability of defect recognition in subsequent detection models, this study applies preprocessing to the raw images acquired during the process. Because the imaging angles and intended applications differ, the top-view and side-view images of potted seedlings exhibit distinct characteristics, requiring separate treatments for different types of noise and geometric features. For the top-view depth images, alignment and background noise removal are performed. Afterward, the depth images are fused with the top-view RGB images, allowing the model to simultaneously consider depth information and defect location. For the side-view images, irrelevant background regions are partially cropped, allowing the model to focus more effectively on detecting defects in the seedlings themselves.

#### 3.2.1. Image Pre-Processing for Top-View Images

Among the defect categories examined in this study, the assessment of leaf shrinkage requires consideration of the relative positional relationship between upper and lower leaves, particularly whether the upper leaves are significantly shorter than the lower ones. To more accurately distinguish leaf layers and positions, RGB-D images with depth information are used as inputs in the top-view analysis, thereby enhancing the model’s ability to recognize the spatial structure of the leaves. Since the RGB and depth images are captured by different sensors, parallax and geometric displacement may occur during imaging. To ensure accurate spatial alignment, geometric correction of the depth image was performed, followed by the removal of extreme background values to reduce noise interference, enabling the model to focus on detecting defects on the seedling leaves.

**Figure 10:**
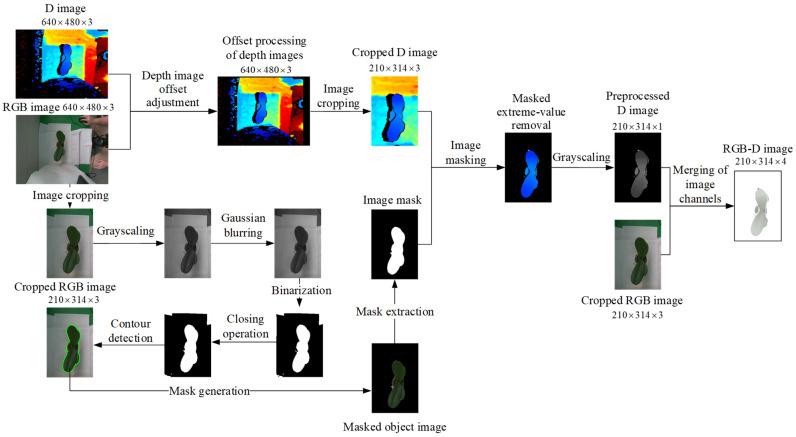
Schematic diagram of top-view image preprocessing.

Figure 10 illustrates the preprocessing workflow applied to the top-view images of potted seedlings. The process begins with capturing both RGB and depth images (640 × 480), followed by offset adjustment of the depth image to align it with the RGB image. After cropping (210 × 314), the RGB image undergoes grayscaling, Gaussian blurring, and binarization to generate a clear plant outline. Contour detection and morphological closing are then applied to refine the mask, which is extracted and overlaid on the seedling to produce the masked object image. In parallel, the cropped depth image is processed with masking and extreme-value removal, then converted to grayscale. Finally, the preprocessed depth and RGB channels are merged to form a four-channel RGB-D image (210 × 314 × 4), which serves as the input for defect detection and feature extraction.

The Intel RealSense D456 camera used in this study can capture RGB and depth images at a resolution of 640 × 480 pixels. At the fixed imaging distance of 58 ± 2 cm, this corresponds to an approximate ground sampling distance (GSD) of 0.36–0.44 mm per pixel, derived from the camera’s intrinsic parameters and field-of-view specifications. Considering sensor noise, depth quantization steps, and lens distortion, the minimum reliably detectable feature is approximately 0.6–0.8 mm. Therefore, small surface defects, such as early-stage anthracnose spots (larger than 1.0 mm), insect bite marks (larger than 2 mm), or leaf shrinkage edges (larger than 1.6 mm), fall within the detectable range of the sensor.

#### 3.2.2. Image Pre-Processing for Side-View Images

Side-view image cropping was performed using a fixed-boundary approach rather than an automated contour-detection method. Because the imaging setup maintained a constant camera position, viewing geometry, and pot placement on the turntable, the spatial distribution of potted seedlings was highly consistent across all samples. To remove irrelevant background while retaining the full plant structure, we empirically determined the cropping borders by visually examining more than 200 annotated images. The final cropping parameters were set to 600 pixels from the left, 650 pixels from the right, and 300 pixels from the top edge of the original 4288 × 2848-pixel images. This approach reliably preserved the entire leaf and root region across all eight viewing angles. Automated contour tracing (e.g., border following or active contour algorithms) was not used because fixed-position imaging produced more stable results without the risk of segmentation noise caused by shadows or pot edges.

### 3.3. Feature Vector Transformation and Labeling

**Figure 11:**
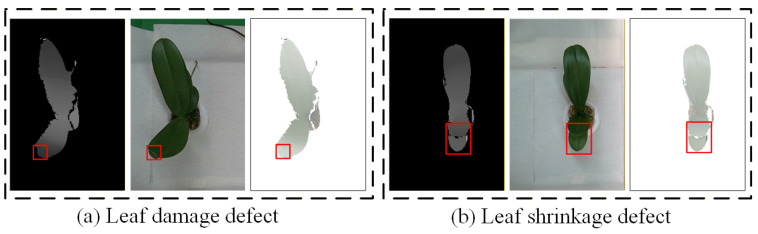
Examples of leaf damage and leaf shrinkage defects in top-view images, including depth, RGB, and fused RGB-D representations (from left to right).

Since the presence of certain defects on the upper leaves affects the subsequent determination of quality grades, this stage focuses on using top-view images to identify whether the upper leaves exhibit leaf damage defects. At the same time, because side-view images are less sensitive to leaf shrinkage defects, this study employs RGB-D images in the top view to facilitate more accurate detection of leaf shrinkage. Figure 11 presents examples of depth images, RGB images, and RGB-D images of seedlings with leaf damage and leaf shrinkage defects. The RGB-D images enhance the model’s ability to recognize shrinkage defects, which are less detectable in side-view images. Accordingly, this study utilizes top-view images to determine whether the upper leaves contain specified defects such as leaf damage or leaf shrinkage.

**Figure 12:**
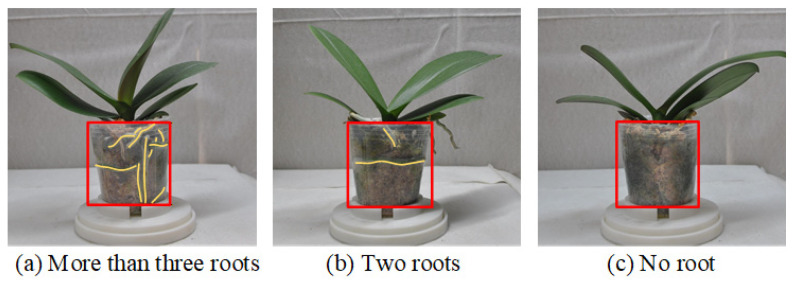
Images of potted seedlings with three different root types in quantities.

The root system grade is determined based on multi-view observations using a systematic set of rules. For each sample, root data from eight viewing angles are consolidated, and the final root grade label is assigned according to the criteria listed in Table 2. Figure 12 illustrates representative images of potted seedlings corresponding to the three different root system grades.

The root grading thresholds in Table 2 follow the practical QC standards used in commercial Phalaenopsis nurseries. Root count alone is insufficient because roots are often partially occluded; therefore, both the number of healthy roots and the number of side-view angles in which they appear are jointly evaluated. The thresholds (e.g., ≥3 roots visible in ≥7 views) correspond to industry benchmarks for root vigor, where more frequent visibility across views indicates a well-distributed, pot-filling root system. These criteria were validated through expert consultation and reflect operational grading practices rather than arbitrary choices.

Finally, the quality grade labels of the samples are assigned manually based on a thorough evaluation of several features. The reference criteria include the number of annotated defects, root quantity, weighted defect areas, and the presence of specific defects in the top-view images. Based on these combined factors, the samples are categorized into three quality grades: Grade A, indicating the highest quality; Grade B, indicating medium quality; and Grade C, indicating the lowest quality.

### 3.4. Proposed Three-Stage Grading Method in This Study

**Figure 13:**
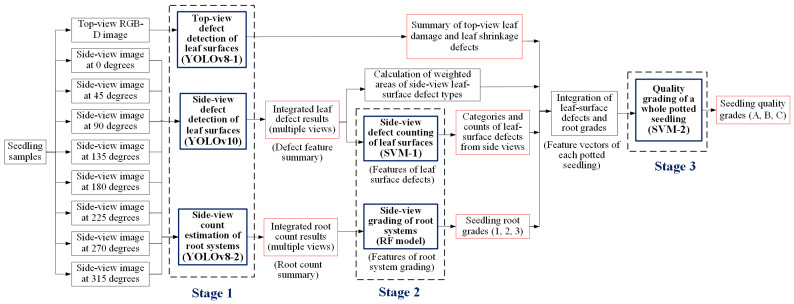
The proposed three-stage grading approach in this study, with red squares indicating outputs of corresponding models.

The grading system developed in this study consists of three stages. Stage 1 includes top-view leaf defect detection, side-view leaf defect detection, and side-view root quantity estimation, each performed using three independent YOLO models to detect defect categories and root counts. Stage 2 involves estimating the number of side-view leaf defects and determining root grades, where an SVM model is applied for defect count estimation and an RF model is used for root grading. Stage 3 performs overall seedling quality grading, in which the final quality grade is determined using an SVM model. Figure 13 shows the proposed three-stage grading approach in this study. The following sections describe the model architectures and training procedures applied in each stage.

#### 3.4.1. YOLOv8 Configuration with Four-Channel RGB-D Image Input

**Figure 14:**
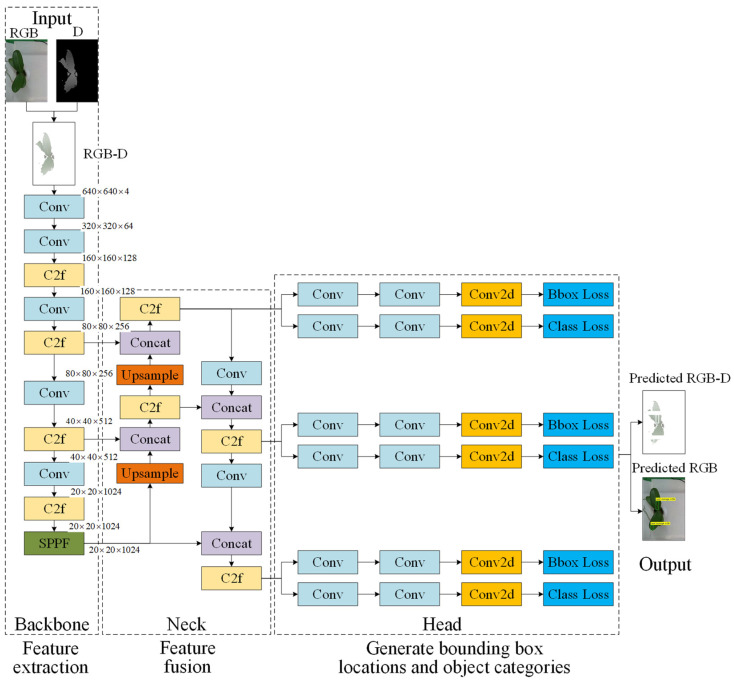
YOLOv8 model architecture for top-view leaf defect detection.

YOLOv8 model [44] and YOLOv10 architecture [45] are adopted for defect and root detection due to their high efficiency, real-time inference capability, and improved feature extraction performance in complex plant imaging conditions. In this study, the YOLOv8 architecture is extended by incorporating depth information as a fourth channel alongside RGB inputs, resulting in an RGB-D configuration. This design enables the model to simultaneously capture surface texture, color variation, and spatial structural features of the leaves, which are particularly important for detecting defects such as shrinkage. Figure 14 illustrates the YOLOv8 model architecture configured with a four-channel RGB-D input for top-view leaf defect detection. The input consists of fused RGB and depth images, enabling the model to capture both color-texture features and spatial-depth information. The architecture follows the standard YOLOv8 design, comprising three main components: the backbone, which extracts multi-scale visual features; the neck, which fuses these features through convolution, upsampling, and concatenation to enhance contextual representation; and the head, which generates bounding box coordinates and class probabilities for defect localization and classification. By incorporating depth as an additional channel, the model enhances its ability to detect defects that depend on leaf structure and relative positioning, thereby improving accuracy in complex cases, such as leaf shrinkage.

To prepare the RGB-D images for YOLO training, the original RealSense depth images (640 × 480) were first aligned with the RGB images and cropped to retain only the seedling region (210 × 314). The resulting regions were then uniformly resized to 640 × 640 pixels, which is the standard YOLO input resolution. The smaller patch shown in Figure 10 (210 × 314 pixels) represents an illustrative zoom-in of a defect area for visualization purposes only and is not used as input to the detection model.

#### 3.4.2. YOLOv10 Configuration for Multi-View Images Using Transfer Learning

Compared with YOLOv8, the YOLOv10 backbone introduces shortcut-based downsampling (SCDown) modules, which enhance feature representation by preserving more spatial information during resolution reduction. The network comprises three key components of YOLO: a backbone for feature extraction, a neck for multi-scale feature fusion, and a head for bounding box regression and classification. However, it utilizes these components to enhance performance on complex, multi-view inputs. To accelerate convergence and improve generalization, pretrained weights from the COCO dataset are adopted as initialization, applying transfer learning to adapt the model to the orchid seedling dataset. This integration of multi-view image analysis, architectural refinements, and pretrained initialization provides stronger detection accuracy than the YOLOv8-based configuration.

In summary, YOLOv8 is best suited for top-view RGB-D images, where depth information provides critical cues for identifying structural defects such as leaf shrinkage. By contrast, YOLOv10 is optimized for multi-view RGB inputs, leveraging SCDown modules and transfer learning to enhance robustness when defects may only be visible from certain angles. Together, these complementary configurations allow the system to adapt to different imaging setups—YOLOv8 excelling in depth-enhanced structural analysis, and YOLOv10 providing comprehensive defect coverage across multiple perspectives.

#### 3.4.3. YOLOv8 Model for Estimating Root Quantity

For root quantity estimation, this study employs a YOLOv8 model trained on side-view RGB images captured from eight different rotational angles around each seedling. The side-view inputs provided comprehensive coverage of the root system, allowing the model to detect and estimate visible roots even when partially occluded in certain views. By utilizing YOLOv8’s efficient feature extraction, multi-scale fusion, and bounding box prediction, the model is able to classify root quantity into predefined categories. Transfer learning with pretrained weights from the COCO dataset is used to accelerate training convergence and improve generalization. The outputs from the eight side-view images are later integrated to generate a more robust and reliable root quantity estimate for each seedling.

#### 3.4.4. SVM Model for Estimating the Number of Leaf-Surface Defects in Potted Seedlings

**Figure 15:**
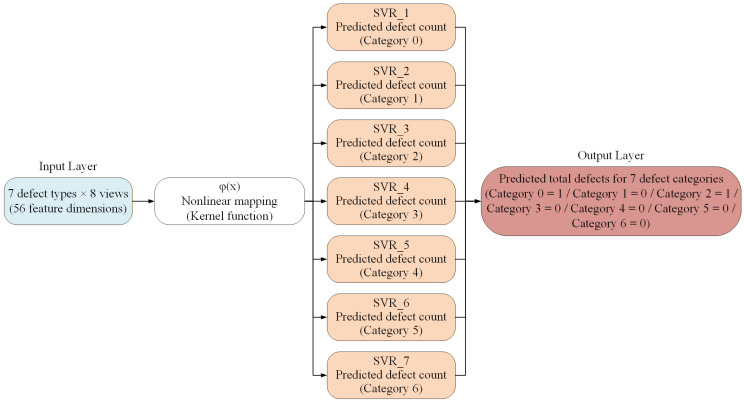
Architecture of the SVM model for estimating leaf-surface defect counts from side-view images of potted seedlings.

The SVM algorithm [46] was employed for classification tasks due to its strong generalization ability in high-dimensional spaces. After the side-view leaf defect detection model completes defect identification, the outputs from the eight viewing angles are further integrated into a consolidated set of leaf defect features. This integrated information is then transformed into feature vectors suitable for processing by machine learning models and subsequently input into an SVM model to estimate the total number of leaf defects for each seedling. Figure 15 illustrates the architecture of the SVM-based model for estimating leaf-surface defect counts from side-view images of potted seedlings. The input layer consists of 56 feature dimensions, derived from seven defect categories across eight side-view images. These features are transformed through a nonlinear kernel mapping function to capture complex relationships. The architecture employs seven independent support vector regression (SVR) units, each dedicated to predicting the defect count of a specific category (Categories 0–6). The output layer then aggregates the predictions to provide the estimated total defect counts for all defect categories, enabling a comprehensive evaluation of seedling leaf-surface conditions.

#### 3.4.5. RF Model for Determining the Root System Grade of Potted Seedlings

**Figure 16:**
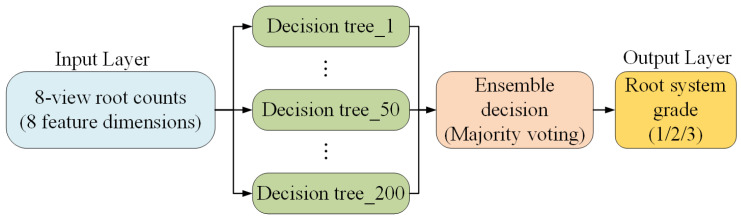
RF model architecture for root system grading of potted seedlings.

The RF model [47], an ensemble-based approach, is utilized for multi-class grading due to its robustness and ability to handle complex feature interactions. After the side-view root quantity estimation model completes the count predictions for each image, the outputs from the eight viewing angles are integrated into a consolidated set of root quantity features. This integrated information is then transformed into feature vectors suitable for machine learning models and input into multiple decision trees. The final root system grade for each seedling is determined using a majority voting approach across the predictions. The architecture of the RF model is utilized for grading the root systems of potted seedlings. Figure 16 presents the architecture of the RF model used for root system grading of potted seedlings. The input layer consists of eight feature dimensions corresponding to root quantity estimates obtained from eight side-view images. These features are processed by multiple decision trees, each independently predicting a root system grade. The outputs of the decision trees are then aggregated through an ensemble decision process using majority voting, which yields the final root system grade label (Grade 1, 2, or 3) in the output layer. This ensemble approach enhances robustness by reducing the influence of individual misclassifications and improving overall prediction stability.

#### 3.4.6. SVM Model for Grading the Quality of Whole Potted Seedlings

**Figure 17:**
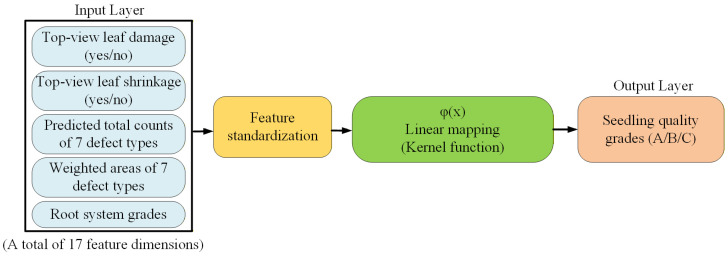
SVM model architecture for whole-seedling quality grading.

After completing the side-view defect count estimation and root system grading, the system further calculates the weighted area of each defect type to serve as an indicator of defect severity. In addition, based on the top-view leaf defect detection results, the system determines whether specific types of defects are present in the sample. The integrated information is then transformed into feature vectors suitable for machine learning processing and used as inputs to an SVM model to predict the overall quality grade of each seedling. Figure 17 illustrates the architecture of the SVM model used for whole-seedling quality grading. The input layer consists of 17 feature dimensions, including the presence or absence of top-view leaf damage and shrinkage, predicted total counts of seven defect categories, weighted areas of the defect types, and the root system grade. These features are first standardized and then processed through a linear kernel mapping function to account for class imbalance. Finally, the output layer produces the predicted overall quality grade of the seedling, classified into three categories: A, B, or C. An example of how raw defect-detection outputs and root-count predictions are converted into the structured feature vector used by the SVM classifier is summarized in Supplementary Example S1.

#### 3.4.7. Mathematical Definition of Defect Weighting and Grade Boundaries

To improve the interpretability of the three-stage grading process, the weighted defect score *S* is now defined mathematically as follows:

(1) $$S=\sum_{i=1}^{k}w_{i}\times A_{i}$$

where *A_i_* represents the total predicted area of defect type *i* (integrated across eight side-view images), *w_i_* is the expert-assigned weight reflecting the relative severity of each defect category, and the variable *k* denotes the number of defect categories. Weight values are determined in consultation with quality-control specialists from collaborating orchid farms and are consistent with practical grading guidelines.

The final quality label is obtained by combining the weighted defect score *S* and the predicted root grade *G*. The A/B/C boundaries are established using calibration against total expert-labeled seedlings and are defined as:

Grade A: *S* < *T_A_* or good quality grade (*G* = 3),
Grade B: *T_A_* ≤ *S* < *T_C_* or moderate quality grade (*G* = 2),
Grade C: *S* ≥ *T_C_* or poor quality grade (*G* = 1),

where *T_A_* and *T_C_* denote score thresholds determined via cross-validation using expert IQC records. These explicit equations make the decision structure fully transparent and traceable.

### 3.5. Direct Grading Method in This Study

**Figure 18:**
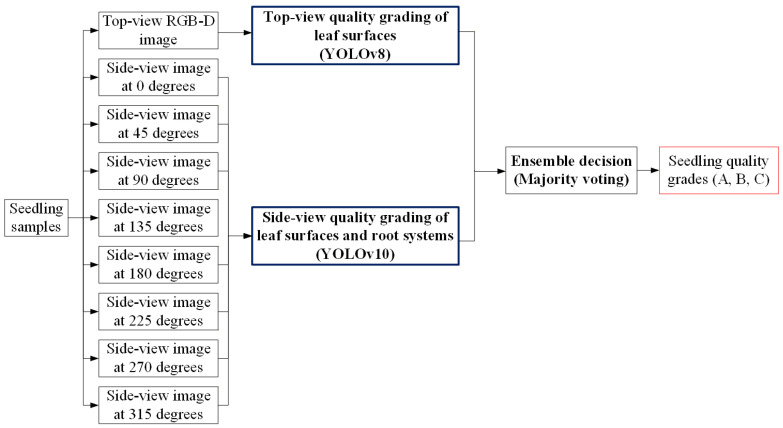
System architecture of the direct grading method.

The three-stage grading method proposed in this study requires sequential processing through three stages to produce the final quality grade, resulting in relatively longer processing times. To address this limitation, an alternative direct grading method was also developed, which requires only a single stage of processing and more closely resembles the current manual visual inspection approach. Figure 18 shows the system architecture of the direct grading method, which streamlines the grading process into a single stage. The system takes both a top-view RGB-D image and eight side-view RGB images of each seedling as input. The top-view image is analyzed using a YOLOv8 model to grade leaf-surface quality, while the side-view images are processed by a YOLOv10 model to assess both leaf-surface defects and root system conditions. The outputs from the two models are then combined through an ensemble decision process using majority voting, resulting in the final seedling quality grade (A, B, or C). This approach provides a faster alternative to the three-stage method while maintaining grading accuracy.

## 4. Results and Discussion

To verify the feasibility of the proposed method for defect detection and quality grading of 2.5-inch Phalaenopsis seedlings, practical experiments and performance evaluations are performed. The experimental results help determine whether the proposed approach can achieve the expected effectiveness in detecting defects and grading quality. Additionally, the performance is compared with that of alternative methods, and a sensitivity analysis is conducted to examine factors that may significantly impact the system.

### 4.1. Experimental Hardware, Captured Images, and User Interface

The hardware equipment used in this study can be divided into two parts: model computation equipment and image acquisition equipment. The model computation equipment consisted of a personal computer with the following specifications: CPU: Intel(R) Core(TM) i7-10700F @ 2.90 GHz, 32 GB RAM, GPU: NVIDIA GeForce RTX 3070, and operating system: Windows 10. The image acquisition equipment included a personal computer with CPU: Intel(R) Core(TM) i5-8250U @ 1.60 GHz, 4 GB RAM, GPU: NVIDIA GeForce MX150, and operating system: Windows 10, along with a Nikon D90 digital single-lens reflex (DSLR) camera, an Intel RealSense D456 depth camera, a rotary turntable for positioning the seedlings, and two fluorescent lamps for illumination. A user interface of the automated quality grading system for outsourced Phalaenopsis potted seedlings is illustrated in Supplementary Figure S7.

### 4.2. Dataset Description and Experimental Workflow

A total of 883 Phalaenopsis 2.5-inch potted seedlings were collected from a commercial orchid production facility in Changhua, Taiwan. The dataset includes 692 seedlings with visible defects and 191 defect-free seedlings, ensuring representation of both normal and abnormal growth conditions. The image collection took place over eight months (September 2024–April 2025) and spanned three commercial greenhouses with varying ventilation, humidity, and lighting conditions, enabling the dataset to capture realistic production variability.

The full dataset covers seven categories of leaf defects, three root system levels, and three seedling quality grades; the complete category distributions are provided in Supplementary Tables S1–S3. All annotations were performed by two trained horticultural specialists, with discrepancies resolved by a senior expert. Inter-annotator agreement was quantified using Cohen’s kappa, yielding κ = 0.87 for leaf defect labeling and κ = 0.91 for root visibility across multi-view images, indicating excellent consistency.

The first stage of the experiments in this study involved parameter setting for the proposed object detection model and regression prediction approach. By adjusting various parameters, the optimal parameter combinations and detection performance were obtained. The second stage consisted of large-sample experiments to select the most effective models for each step. The dataset is organized at the seedling level with the required number of samples for each step listed in Supplementary Table S4. The third stage integrated the selected models into a complete system, and the overall system performance was evaluated. Finally, the fourth stage conducted sensitivity analyses to examine the robustness of the proposed method, focusing on the influence of environmental lighting variations, repeated defect detections across multiple viewing angles, and the impact of different viewing perspectives on overall system performance.

### 4.3. Performance Evaluation Indices

In this study, different performance indicators are used based on each model’s specific functions. The model functions are divided into three main categories: defect detection, quantity estimation, and quality grading. For quantity estimation, two different models are applied to perform separate estimation tasks. In the first stage, the side-view root count estimation used the YOLO model to detect and estimate root numbers from images, with results classified into three predefined quantitative ranges. In the second stage, the leaf defect count estimation for potted seedlings utilized an SVM model, which combined defect type and count information from eight side-view images to predict the overall defect types and quantities for each seedling. Unlike the interval-based results of the YOLO approach, the SVM-based method provided specific numerical predictions rather than categorical ranges.

Therefore, when selecting performance metrics, if the model output is a discrete category or grade, the classification and grading performance indicators listed in Table 3 are used, according to Equations (2)–(7). Conversely, if the model output is a continuous numerical value, the regression performance indicators shown in Table 4 are applied, corresponding to Equations (8)–(10).

(2) $${Precision}_{i}=P_{i}=\frac{{TP}_{i}}{{TP}_{i}+{FP}_{i}}\times 100\%$$

(3) $${Recall}_{i}=R_{i}=\frac{{TP}_{i}}{{TP}_{i}+{FN}_{i}}\times 100\%$$

(4) $${F1\text{-}score}_{i}={F1}_{i}=\frac{2\times {Precision}_{i}\times {Recall}_{i}}{{Precision}_{i}+{Recall}_{i}}\times 100\%$$

(5) $$\mathrm{Overall}\text{\_}\mathrm{Precision}=\frac{\sum_{i}n_{i}\cdot P_{i}}{\sum_{i}n_{i}}$$

(6) $$\mathrm{Overall}\text{\_}\mathrm{Recall}=\frac{\sum_{i}n_{i}\cdot R_{i}}{\sum_{i}n_{i}}$$

(7) $$\mathrm{Overall}\text{\_}F1\text{-}\mathrm{score}=\frac{\sum_{i}n_{i}\cdot {F1}_{i}}{\sum_{i}n_{i}}$$

Table 3 defines the precision, recall, and *F1-score* metrics used to evaluate the performance of the proposed classification and grading models at both the category level and the overall system level. Table 4 defines the Mean Absolute Error (*MAE*), Root Mean Squared Error (*RMSE*), and Coefficient of Determination (*R*^2^) used to evaluate the predictive accuracy of the regression models developed in this study. Lower MAE and RMSE values indicate higher prediction precision, while higher R^2^ values reflect stronger model fitting performance.

(8) $$MAE\;\left(Mean\;Absolute\;Error\right)=\frac{1}{n}\sum_{j=1}^{n}\left|y_{j}-{\hat{y}}_{j}\right|$$

(9) $$RMSE\;(Root\;Mean\;Squared\;Error)=\sqrt{\frac{1}{n}\sum_{j=1}^{n}{\left(y_{j}-{\hat{y}}_{j}\right)}^{2}}$$

(10) $$R^{2}Score=1-\frac{\sum_{j=1}^{n}{\left(y_{j}-{\hat{y}}_{j}\right)}^{2}}{\sum_{j=1}^{n}{\left(y_{j}-{\bar{y}}_{j}\right)}^{2}}$$

### 4.4. Parameter Settings of Deep Learning and Machine Learning Models

To enhance the accuracy and generalization of the proposed defect and root detection models, parameter optimization is conducted for both deep learning and machine learning frameworks. For YOLO models, key hyperparameters such as the optimizer, learning rate, and batch size are tuned, while SVM and RF models are optimized based on their respective kernel and tree parameters. Model performance is evaluated using the *R*^2^ score and the *Overall_F1-score* to determine the optimal parameter combination that yields the best detection accuracy. The detailed configurations are presented in Supplementary Tables S5 and S6.

### 4.5. Selecting YOLO Models for Leaf Defect Detection and Root Count Estimation

**Figure 19:**
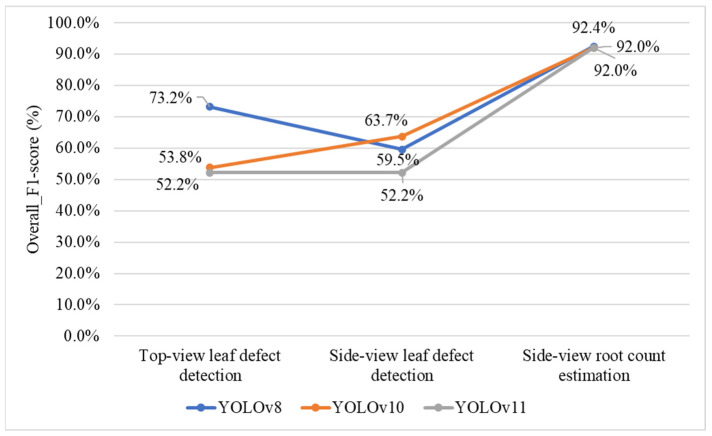
Effectiveness comparison of YOLO models for leaf defect detection and root count estimation in stage 1.

To determine the most suitable YOLO model for defect detection and root system estimation in Stage 1 of the proposed grading framework, Figure 19 compares the effectiveness of three YOLO variants, YOLOv8, YOLOv10, and YOLOv11, across three detection tasks: top-view leaf defect detection, side-view leaf defect detection, and side-view root count estimation. Among these models, YOLOv8 achieves the highest *Overall_F1-scores* in both top-view leaf defect detection (73.2%) and side-view root count estimation (92.4%), demonstrating its superior capability in identifying diverse leaf surface defects under varied imaging conditions. For root count estimation, all three models perform comparably, with *F1-scores* exceeding 92%, indicating high reliability in detecting root structures. Based on these findings, YOLOv8 is selected as the optimal model for subsequent stages, as it provides the best balance between detection accuracy and overall stability across all tasks.

### 4.6. Selecting Machine-Learning Models for Leaf Count Estimation and Quality Grading

**Figure 20:**
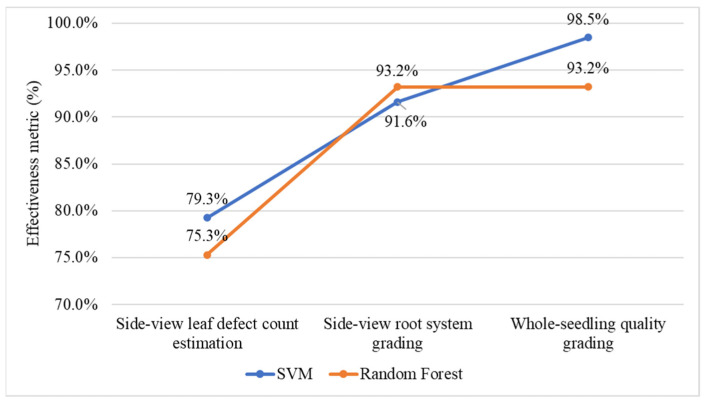
Effectiveness comparison of machine-learning models for defect count estimation and quality grading in stages 2 and 3.

To identify the most suitable machine learning models for estimating leaf defect count, grading root systems, and classifying overall seedling quality in Stages 2 and 3 of the proposed grading system, Figure 20 compares the effectiveness of two algorithms, SVM and RF, across these three tasks. For side-view leaf defect count estimation, SVM achieves slightly higher accuracy (79.3%) than RF (75.3%), indicating better robustness in handling small and complex defect patterns. In root system grading, both models perform well, with RF attaining a marginally higher effectiveness metric (93.2%) compared to SVM (91.6%). However, for whole-seedling quality grading, SVM achieved the highest performance with an effectiveness score of 98.5%, demonstrating superior capability in integrating multi-dimensional feature vectors for final quality classification. Overall, SVM exhibits greater stability and accuracy across tasks and is therefore selected as the optimal model for defect count estimation and quality grading in subsequent analyses.

### 4.7. Performance Evaluation of Three-Stage and Direct Grading Methods in This Study

These three stage-specific models are then integrated to evaluate the overall system performance. Table 5 presents the performance indicators, including *F1-score* and *R^2^* value, of the optimal model combinations selected at each stage of the proposed three-stage grading method. In Stage 1, YOLOv8 achieves 73.20% for top-view leaf defect detection, while YOLOv10 performs slightly lower at 63.70%, and YOLOv8 performs strongly for side-view root count estimation with 92.40%. In Stage 2, SVM-1 is applied for estimating the side-view leaf defect count, yielding an R^2^ value of 0.7026. Meanwhile, the RF model achieves an accuracy of 89.43% for root system grading. Finally, in Stage 3, SVM-2 provides the highest performance, achieving an *F1-score* of 80.43% for whole-seedling quality grading. These results indicate that the performance of side-view leaf-surface defect detection in the first stage directly affects the accuracy of defect count estimation in the second stage. However, in the third stage, seedling quality grading is determined through the integration of multiple feature vectors, which effectively reduces the impact of errors from the previous stages. Ultimately, the three-stage grading method achieves an *Overall_F1-score* of 80.43% in the final output of seedling quality grading, demonstrating that the proposed system can effectively perform seedling quality classification and has practical applicability.

### 4.8. Misclassification Analyses and Failure Cases

#### 4.8.1. Misclassification Cases of Single-Sided Leaf Defect Detection in Stage 1

To analyze the misclassification cases of the more complex Stage 1 side-view leaf defect detection model, we generated a category-wise confusion matrix (Supplementary Table S7) that summarizes how each leaf defect type was predicted. Table 6 presents the performance indicators for category-wise classification in single side-view images. The results show that the “Flawless” category achieves a relatively high recall of 86% but lower precision (64%), indicating a high rate of false positives. Disease and insect damage categories perform poorly, with recall values of 44% and 20%, respectively, suggesting that many true defect cases are missed. In contrast, categories such as leaf shrinkage (81% *precision*, 66% *recall*, *F1-score* 72%), variation (84% *precision*, 70% *recall*, *F1-score* 77%), and lower-leaf yellowing (80% *precision*, 77% *recall*, *F1-score* 78%) demonstrate more balanced and reliable classification. Overall, the analysis highlights that pest damage and disease detection are the most challenging for the model, whereas variation and lower-leaf yellowing are identified with higher robustness.

As shown in the detailed per-class evaluation, the detection of disease-related and pest-related defects exhibited comparatively lower recall values, primarily due to (1) fine-scale symptoms with subtle color variation, which are difficult for bounding-box detectors to localize precisely; (2) occlusion by overlapping leaves, especially in early-stage disease infections; and (3) class imbalance, since disease and pest damage occur less frequently in commercial production, resulting in limited training samples. In addition, severe backlighting or shading in certain viewing angles occasionally reduced model confidence, increasing the likelihood of missed detections.

Although the disease and pest-damage detection performance is relatively weak when evaluated on single side-view images, the proposed grading framework does not rely on a single viewpoint. Instead, the system captures eight distinct viewing angles, and the final defect determination is made by consolidating detections across all views. A seedling is considered to exhibit a particular defect type if any one of the eight perspectives successfully detects that category. This multi-view aggregation substantially enhances overall recall by compensating for occlusions, viewpoint-dependent visibility, and low-contrast issues that may not be consistently visible across all angles. As a result, even though per-view recall for disease and insect damage is limited, the multi-view fusion strategy effectively increases the combined detection sensitivity, reduces the probability of missed detections, and preserves the practical utility of the system for real-world orchid seedling inspection and grading.

#### 4.8.2. Failure Case Analyses in Final Quality Grading

To provide a more comprehensive evaluation of the proposed grading system, we conducted a detailed error analysis that includes confusion matrices and per-class performance metrics for the three final quality grades (A, B, and C). Table 7 and Table 8 present the confusion matrices for both the three-stage grading method and the direct grading method, while Table 9 reports per-class *precision*, *recall*, and *F1-scores* for both methods.

The three-stage method achieved an overall *F1-score* of 0.8043, with class-wise *F1-scores* of 0.6512 (A), 0.8705 (B), and 0.7164 (C). In contrast, the direct grading method yielded a higher overall *F1-score* of 0.8916, along with improved *F1-scores* for Grades A (0.7380), B (0.9464), and C (0.8333).

Analysis of the confusion matrices provides deeper insight into the behavior and practical reliability of the two grading strategies. Although the three-stage method achieves a slightly lower overall *F1-score* than the direct method, it produces fewer extreme cross-category errors. Specifically, it misclassifies 2 Grade-A seedlings as Grade-C and 3 Grade-C seedlings as Grade-A, whereas the direct method results in 3 A→C and 4 C→A misclassifications. These severe misgrading cases are particularly critical in commercial orchid production because confusion between Grade A and Grade C directly affects contractor compensation and compromises the assurance of export-quality seedlings. The results, therefore, highlight that, despite its marginally lower aggregate accuracy, the three-stage method offers more stable and risk-averse performance—an essential property for real-world quality control workflows.

The comparative analysis of the two grading strategies reveals complementary strengths. The three-stage workflow enhances interpretability by decomposing the decision process into three distinct stages: (1) defect detection, (2) object quantification, and (3) final grade integration. These intermediate supervisory steps constrain the decision pathway and appear to reduce large misclassification jumps between quality categories (e.g., A↔C), even though its overall *F1-score* is slightly lower than that of the direct approach. In contrast, the direct grading method optimizes end-to-end predictive accuracy without intermediate structural constraints, enabling higher aggregate performance but increasing susceptibility to occasional large semantic shifts in predicted quality. These findings validate our design rationale: the three-stage system should remain the primary, explainable decision pipeline for operational grading, while the direct method is best used as a rapid triage tool for high-throughput screening, with downstream confirmation via the interpretable three-stage workflow when needed.

### 4.9. Robustness Analysis of the Proposed Method

This section primarily addresses the issues of defect detection and multi-view defect counting, while also examining the importance of each viewing angle for the model. The discussion encompasses several aspects: the impact of varying environmental lighting conditions on defect detection performance, whether the same defect can be counted repeatedly from different viewing angles, the contribution of each view to the overall system effectiveness, and the influence of modifying the Stage 1 model on the entire system. Additionally, the effects of using different types of top-view images on the system are examined. Through sensitivity analysis, the robustness of the proposed method is further evaluated.

#### 4.9.1. Impact of Environmental Lighting Changes on Detection Performance

To examine how different environmental lighting conditions influence detection performance, this study simulates various illumination levels through linear brightness adjustments. The adjustment method applied a brightness coefficient (α) as a linear scaling parameter, with values set to 0.25, 0.5, 0.75, 1.25, 1.5, and 1.75. When α = 1, the image remains unchanged from its original brightness; when α < 1, the overall brightness of the image is reduced compared to the original; and when α > 1, the overall brightness of the image is increased relative to the original. The brightness adjustment formula is expressed in Equation (10), where *I(x,y)* represents the original pixel value, and *B_α_(x,y)* denotes the pixel value after brightness adjustment.

(11) $$B_{\alpha }\left(x,y\right)=\alpha \cdot I\left(x,y\right)$$

**Figure 21:**
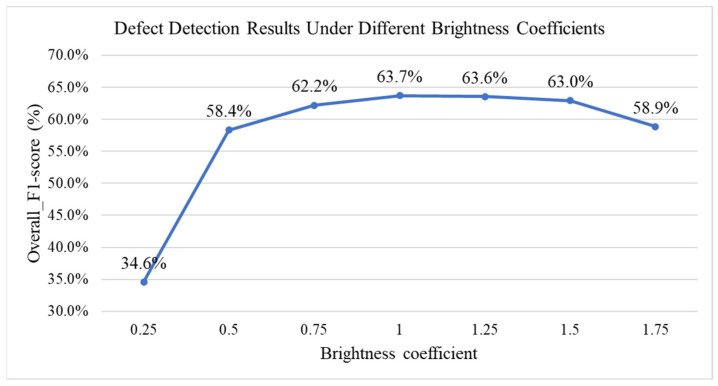
Detection performance results under different brightness coefficients.

Figure 21 presents the detection performance results under different brightness coefficients. The results indicate that when α is set to 0.25, the model’s performance decreases significantly, demonstrating that excessively low environmental brightness has a negative impact on side-view leaf defect detection. It is therefore recommended that users avoid conducting seedling defect detection under extreme lighting conditions in practical applications.

#### 4.9.2. Impact of Repeatedly Counting the Same Defect from Different Viewpoints on Detection Performance

**Figure 22:**
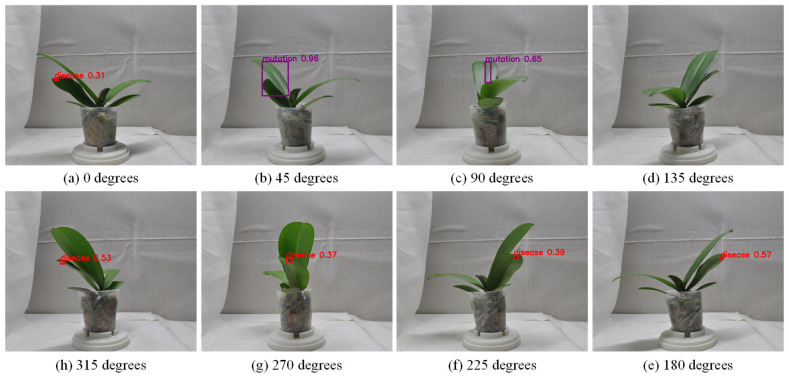
Defect detection result images of the same potted seedling captured from 8 viewing angles.

This section investigates whether repeated detections of the same defect across multiple viewing angles in the Stage 1 side-view leaf defect detection model affect the accuracy of defect count estimation in Stage 2, potentially leading to significant discrepancies between predicted and actual defect counts. Figure 22 presents an example where the same defect is detected across several views, confirming that duplicate detections occur under multi-view conditions. To clarify this issue, this study adjusts the number of viewing angles and compares the performance of defect count estimation under multi-view versus fewer-view settings. This analysis investigates whether increasing the number of views results in repeated defect counts that affect estimation outcomes, thereby evaluating the stability and reliability of the proposed defect count estimation method under various viewing conditions.

**Figure 23:**
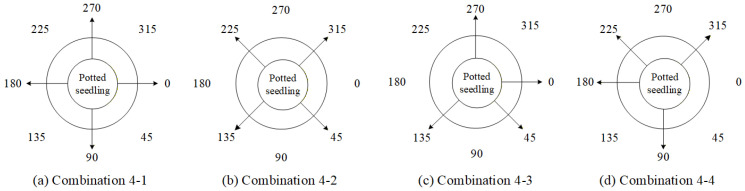
Four combination types of removing four viewing angles from the eight side views.

In this study, four types of 4-view combinations were first designed and compared with the full 8-view approach to analyze the performance of defect count estimation and investigate the impact of the number of viewing angles on the estimation results. The 4-view combinations are shown in Table 10, and Figure 23 illustrates the schematic arrangement diagrams of the corresponding viewing ranges for each combination. In the subsequent Table 10, Table 11, Table 12, Table 13 and Table 14, a checkmark indicates that the angle has been selected.

**Figure 24:**
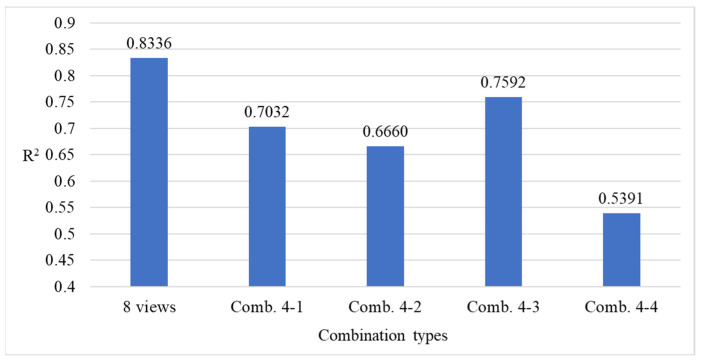
Comparison of model performance using all viewing angles and four four-view combinations.

Figure 24 presents the comparison results of model performance using all viewing angles versus the four combinations of four viewing angles. The results indicate that utilizing all viewing angles provides estimates closer to the actual defect count, while combination 4-3 achieves better performance among the four-view combinations, despite using only four angles.

**Figure 25:**
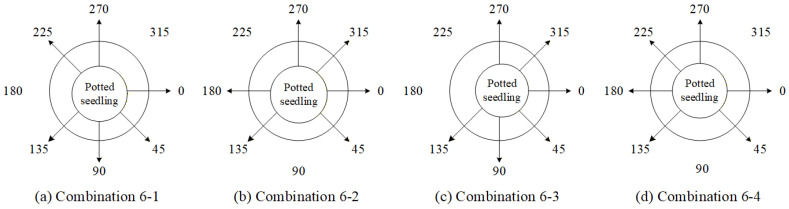
Four combination types of removing two viewing angles from the eight side views.

Since combination 4-3 achieved relatively good performance with fewer viewing angles, this study further adds two more angles to design four types of six-view combinations. These are then compared across all viewing angles to analyze the performance of defect count estimation and to examine the impact of the number of viewing angles on the estimation results. The six-view combinations are listed in Table 11, and Figure 25 illustrates the schematic ranges of the corresponding viewing angles for each combination.

**Figure 26:**
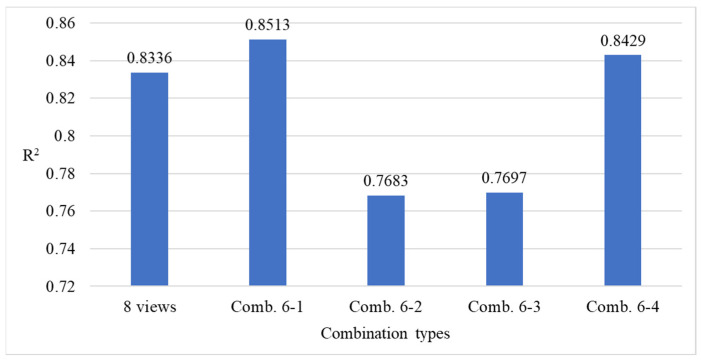
Comparison of model performance using all viewing angles and four six-view combinations.

Figure 26 presents the performance comparison results between using all viewing angles and the four six-view combinations. The results indicate that combinations 6-1 and 6-4, despite excluding two viewing angles, produced prediction results that were closer to the actual defect counts than those obtained using all eight views.

This study investigates whether multi-view detection could affect prediction accuracy due to repeated counting of the same defect by comparing the performance of defect count estimation under different numbers of viewing angles. The experimental results show that when using 6 to 8 views, the estimated results are closer to the actual defect counts, with relatively smaller errors. In contrast, when fewer views are used, prediction errors increase significantly. These findings indirectly demonstrate that the defect count estimation method proposed in this study does not suffer from increased estimation errors due to repeated detection of the same defect when the number of views increases. Instead, the additional views provided more comprehensive defect information, thereby improving overall accuracy. Therefore, this study recommends using 6 to 8 viewing angles as the optimal range.

#### 4.9.3. Ablation Experiments to Evaluate How Various Viewpoint Combinations Influence Overall System Performance

**Figure 27:**
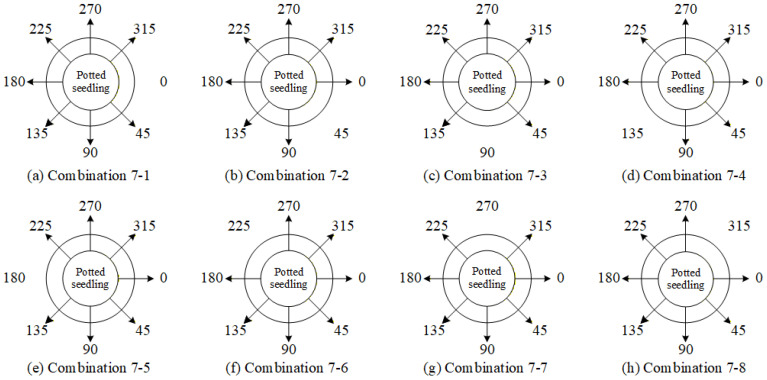
Eight combination types of removing one viewing angle from the eight side views.

This section conducts an ablation experiment by gradually reducing the number of side-view angles to evaluate the contribution of each view to the overall system performance. First, each side-view image is removed one at a time to observe its impact on the model’s effectiveness and identify the most influential angles. Figure 27 illustrates the eight experimental combinations, each retaining seven views while excluding one specific angle, allowing for a detailed assessment of how the removal of each view affects the accuracy of defect count estimation.

**Figure 28:**
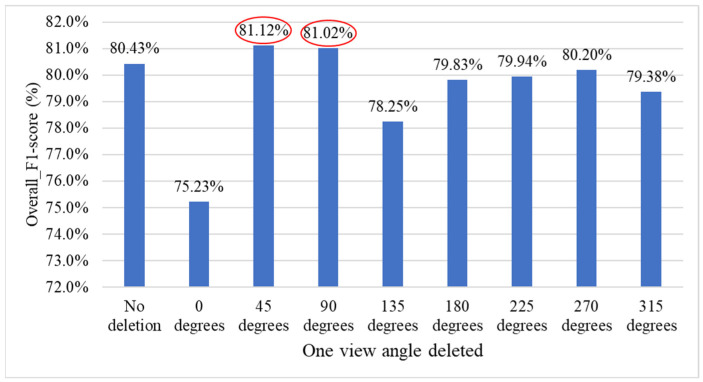
Impact of removing a single side-view angle on the overall grading system performance.

Figure 28 presents the performance comparison between using all viewing angles and removing one side-view angle for the overall grading system. The results show that when the 45° or 90° view is removed, the overall system performance slightly improves compared to using all views (red ellipses).

**Figure 29:**
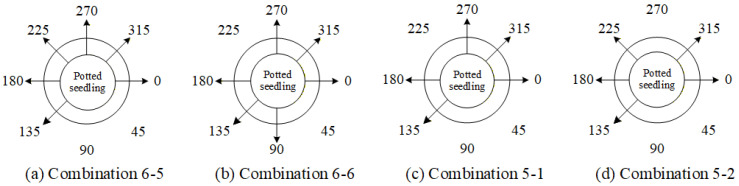
Four combination types of removing two and three viewing angles from the eight side views.

Based on the results of the single-view ablation experiments, several key viewing angles that significantly impact the system performance are identified. The order of deletion priority, determined by their degree of influence on the overall system performance, is 45° > 90° > 270° > 225°. Furthermore, four angle-deletion combinations are designed, as listed in Table 12, with their corresponding viewing positions illustrated in Figure 29.

**Figure 30:**
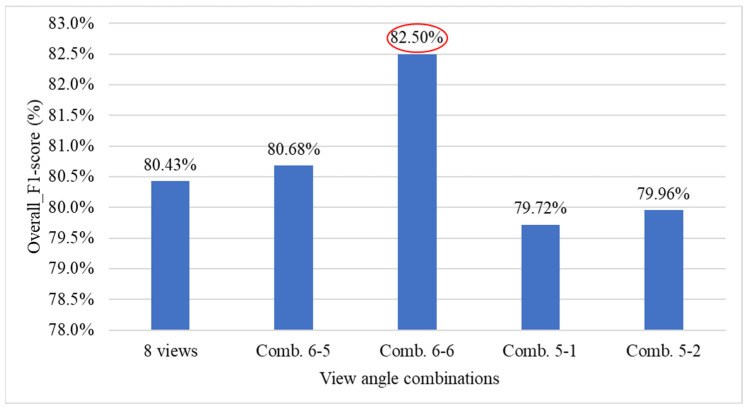
Performance comparison of the grading system under combinations of deleting two and three key viewing angles.

Figure 30 presents the performance comparison results between using all viewing angles and combinations of deleting 2 or 3 viewing angles for the overall system. The results show that with combination 6-6, the overall system performance improved by 2.07% (red ellipse), indicating that simultaneously removing the 45° and 225° views has a significant impact on system performance.

**Figure 31:**
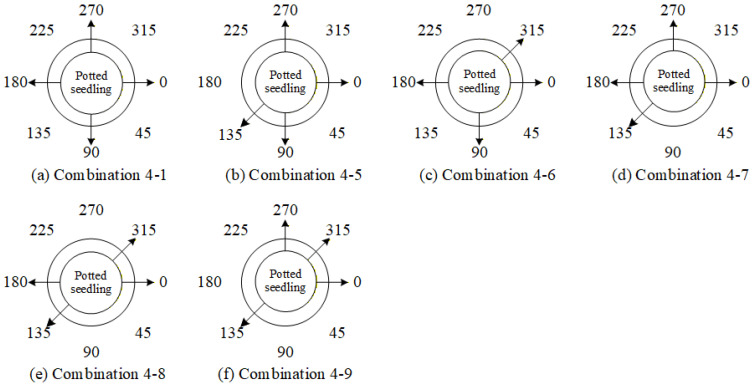
Six combination types of removing four viewing angles from the eight side views.

Based on the previous experiments, it has been found that simultaneously removing the 45° and 225° viewing angles has a significant impact on the overall system. Building on this, the study further explores the effect of removing two additional viewing angles. To avoid leaving large portions of the seedlings undetected, combinations in which more than three consecutive viewing angles are removed were not considered. Moreover, since leaf shrinkage defects can only be effectively detected at 0° and 180°, combinations that exclude the 0° view were also disregarded. Taking these considerations into account, the deletion combinations of four viewing angles were designed as shown in Table 13, with their corresponding positions illustrated in Figure 31.

**Figure 32:**
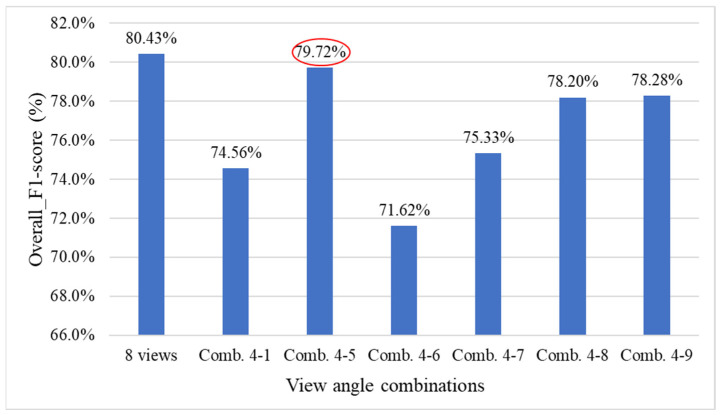
Performance comparison of the grading system under combinations of deleting four viewing angles.

Figure 32 presents the performance comparison results between using all viewing angles and combinations of deleting four viewing angles for the overall system. The results show that with combination 4-5, the overall system performance experienced a smaller decline (red ellipse).

**Figure 33:**
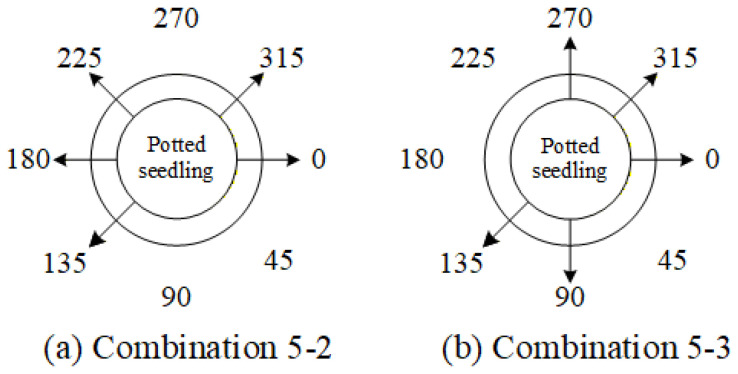
Two combination types of removing three viewing angles from the eight side views.

Based on the previous experiments, it was inferred from combinations 4-5 and 4-9 that simultaneously removing the 45°, 180°, and 225° viewing angles results in a smaller decrease in overall system performance. Therefore, this study compares this combination with combination 5-2 to evaluate their impact on the model’s overall performance. The deletion combinations of three viewing angles are listed in Table 14, and their corresponding viewing positions are illustrated in Figure 33.

**Figure 34:**
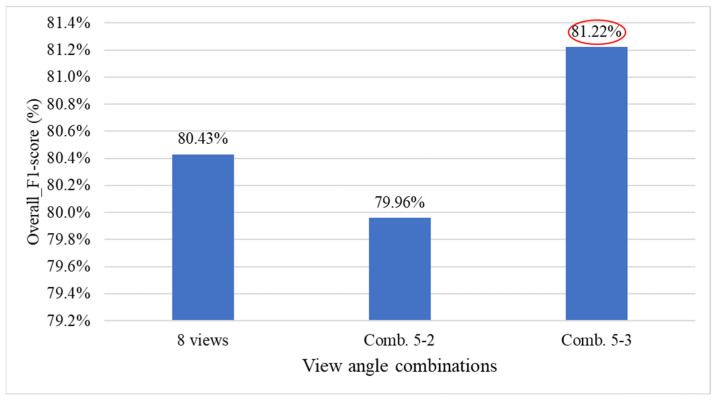
Performance comparison of the grading system under combinations of deleting three viewing angles.

Figure 34 presents the performance comparison between using all viewing angles and deleting combinations of three viewing angles for the overall system. The results show that with combination 5-3, the overall grading system performance slightly improved by 0.79% (red ellipse).

Table 15 presents the optimal combination configurations corresponding to different numbers of deleted viewing angles. Among them, combination 6-6 achieved the best performance, with a weighted *F1-score* of 84.78%. Therefore, in situations where detection must be performed using fewer viewing angles, this study recommends prioritizing the six-view combination that excludes the 45° and 225° angles.

#### 4.9.4. Effect of Changing the YOLO Model in the Defect-Detection Stage on Overall System Performance

To investigate whether using different model combinations in Stage 1 yields better performance than using a single model type across all stages, this study conducts a comparative analysis of various model configurations for both the proposed three-stage grading method and the direct grading method. In the experiments, two high-performing models, YOLOv8 and YOLOv10, are selected for combination testing. Table 16 presents the performance results of the three-stage grading method under different model combinations. The results indicate that when YOLOv8 is used exclusively in Stage 1, the overall system performance is superior. Table 17 presents the performance results of the direct grading method under various model combinations, indicating that the combination of YOLOv8 and YOLOv10 yields the best overall system performance.

**Figure 35:**
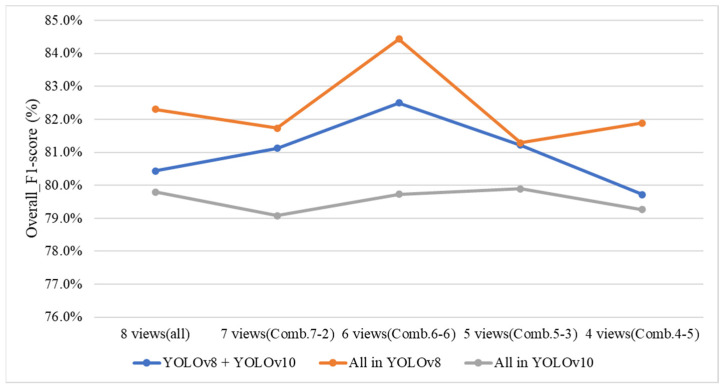
Comparison of *F1-score* performance indicators for four optimal viewing angle combinations and YOLO model combinations in stage 1 of the three-stage grading method.

Figure 35 illustrates the comparison of overall *F1-scores* for different YOLO model combinations and optimal viewing angle configurations in Stage 1 of the three-stage grading method. The results show that using YOLOv8 for all detection tasks achieves the highest performance, reaching an *F1-score* of approximately 84.5% under the 6-view (Comb. 6-6) configuration. In contrast, the combination of YOLOv8 and YOLOv10, along with the configuration that utilizes all 8 views, yields slightly lower *F1-scores*. Meanwhile, the YOLOv10-only setup consistently demonstrates the lowest performance across all view combinations. These results indicate that YOLOv8 offers more stable and accurate detection when fewer, but optimally selected, viewing angles are used.

#### 4.9.5. Effect of Including or Excluding Top-View Images and Top-View Depth (D) Images on Overall System Performance

To investigate the impact of including or excluding top-view RGB and depth (D) images on the grading performance of the three-stage and direct grading methods, this study conducts a comparative experiment using two configurations: the three-stage grading method with YOLOv8 applied in all Stage 1 models, and the direct grading method combining YOLOv8 and YOLOv10. In the experiment, the top-view RGB and depth images are sequentially excluded to observe their effects on grading performance. Additionally, the four optimal viewing angle combinations identified from the previous ablation experiments are used for evaluation.

**Figure 36:**
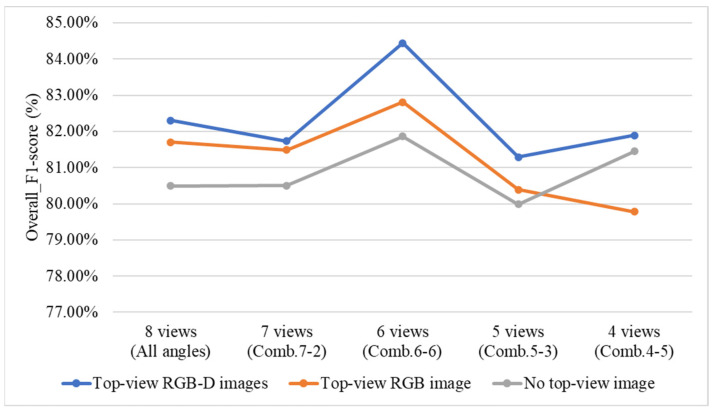
Comparative analysis of the three-stage grading method using three types of top-view images.

Figure 36 compares the performance differences of the three-stage grading method under various types of top-view images. The results indicate that the grading performance is highest when RGB-D top-view images are used. When the top-view depth (D) images are excluded, the performance decreases, and when top-view images are entirely removed, the performance is the lowest among the three conditions.

**Figure 37:**
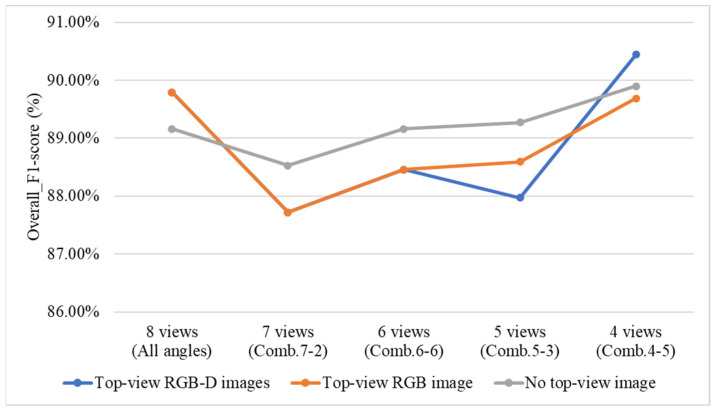
Comparative analysis of the direct grading method using three types of top-view images.

Figure 37 illustrates the performance differences of the direct grading method under various types of top-view images. The results show that when using 8-view and 4-view side images, the performance is highest with RGB-D top-view images. When the top-view depth (D) images are removed, the performance is generally the lowest across most viewing-angle conditions. However, when top-view images are completely excluded, the 7-view, 6-view, and 5-view configurations exhibit better performance.

According to Figure 36 and Figure 37, the performance of the three-stage grading method decreased noticeably when the top-view depth (D) images are not used, whereas the direct grading method shows no significant performance change under the same condition. The results show that the three-stage grading method, when using RGB-D top-view images combined with six side-view angles, achieved an *F1-score* of 84.44%. In contrast, the direct grading method, which utilizes RGB-D top-view images combined with four side-view angles, achieves an *F1-score* of 90.44%. Both methods demonstrate effective performance in the quality grading of Phalaenopsis potted seedlings.

### 4.10. Discussion and Limitations

Although the proposed three-stage grading method serves as the primary workflow in this study due to its interpretability and structured diagnostic process, a complementary direct grading method is also designed to address practical needs in large-scale commercial orchid production. The three-stage method generates intermediate outputs, including leaf-defect detections, defect counts, weighted severity scores, and root system grades, allowing quality inspectors to understand *why* a seedling receives a particular grade. This explainability is essential for resolving contractor–farm disputes, training new inspectors, and meeting traceability requirements in export-quality assessment.

However, real-world greenhouse operations often require rapid screening of thousands of seedlings per day, where processing speed becomes a practical bottleneck. To support high-throughput inspection, the direct grading method uses a single-stage YOLO-based classifier to output the final A/B/C quality grade directly from multi-view imagery. This approach forgoes intermediate interpretive steps and therefore cannot explain the cause of grading outcomes; however, it offers substantially faster inference, enabling operators to quickly flag seedlings that clearly belong to high- or low-quality categories.

In practice, the direct method is intended for initial triage and rapid throughput, while the three-stage method remains the primary, interpretable workflow for samples requiring detailed evaluation, borderline cases, and situations where transparent reasoning is necessary for decision-making or contractor compensation. Together, these two methods provide a balanced grading framework that meets the industry’s demands for both explainability and operational efficiency.

Although the proposed grading system integrates multiple sensing modalities and modeling components (RGB-D top view, multi-view side images, YOLO detectors, SVM/RF classifiers), this design is driven by the practical constraints of Phalaenopsis seedling inspection rather than unnecessary complexity. Seedlings exhibit strong 3D occlusion, and many defects, such as lesions on lower leaf surfaces or roots emerging behind overlapping leaves, cannot be captured from a single viewpoint. Our ablation studies confirm that reducing side-view angles or removing depth information significantly degrades defect detection and, consequently, final grading accuracy.

Furthermore, multiple models are required because the tasks are fundamentally different: YOLO handles instance detection, while SVM and RF are more suitable for multi-view defect aggregation, severity assessment, and final grade classification. Attempts to replace the system with a single end-to-end model produced lower stability and reduced interpretability, particularly for boundary grades that matter most economically. Importantly, despite its internal complexity, the operational workflow remains simple because image capture is automated via a motorized turntable, and inference is fully integrated. Future work will explore more compact multi-task architectures to streamline the pipeline while maintaining current performance levels.

In the current study, YOLO-based detection is selected primarily because of its computational efficiency, ease of deployment in real-time greenhouse applications, and its proven strengths in general horticultural object detection tasks. Since the overall goal of the system is to support high-throughput grading of large seedling batches, the operational simplicity of YOLO models offered significant advantages in terms of system responsiveness and hardware feasibility. However, we acknowledge that for small, fine-grained, or irregular defects, segmentation-based models, such as Mask R-CNN, U-Net, HRNet, or more recent transformer-based approaches like SegFormer, could provide a more precise delineation of lesion regions, contributing to improved recall and more accurate downstream defect quantification.

Recent advances in generative modeling offer a promising path for enhancing defect-detection robustness, particularly given the significant class imbalance observed in this study. Rare defects, such as early-stage disease lesions, mild pesticide injuries, and uncommon morphological anomalies, were underrepresented in our dataset, which limited recall for these categories. Synthetic data augmentation using modern GAN-based methods can help enrich minority classes while preserving realistic horticultural characteristics. The modified WGAN-GP framework [48] has shown excellent performance in generating high-fidelity agricultural RGB/IR imagery with improved structural and textural fidelity. Incorporating such generative augmentation into our YOLO training pipeline could strengthen the detection of rare defect types and reduce reliance on large, manually collected datasets. Future work will therefore explore tailored GAN-based augmentation strategies to rebalance defect classes and further enhance both the three-stage and direct grading systems.

At the current stage, our study focuses on developing and validating the proposed multi-view hybrid grading framework, optimizing the detection and classification models, and analyzing performance through multiple ablation experiments. Because the dataset used in this project was incrementally collected over several production cycles, applying comprehensive statistical confidence analysis would require a more standardized and evenly distributed dataset across defect categories and growth batches. Future work will include additional statistical examinations, such as significance testing between model variants and uncertainty quantification for per-class predictions, to strengthen the robustness claims of the proposed system.

To complement the comparison with manual inspection (average 18 s per seedling), we evaluate the real-time operational efficiency of the proposed automated grading system. In our setup, image acquisition, which includes placing the seedling on the turntable, capturing a top-view RGB-D image, and acquiring eight side-view RGB images, required 10.5 ± 0.5 s. The three-stage grading pipeline (YOLO-based defect/root detection, feature extraction, and SVM/RF inference) required an additional 1.9 ± 0.1 s on an RTX 3070 GPU, resulting in a total processing time of ≈approximately 12.4 s per seedling, a 31% reduction compared with manual inspection. When using only the direct grading method, model inference required 0.84 ± 0.1 s, resulting in a total time of ≈approximately 11.4 s per seedling, making it well-suited for rapid first-stage screening. These results show that the system provides both higher consistency and substantially faster throughput than human graders, even without hardware optimization. Future work will further reduce processing time by utilizing multi-camera configurations and batch-optimized inference.

Based on interviews and feedback from collaborating orchid producers, commercial acceptance of automated grading requires an *overall_F1-score* above approximately 85–90%. Misclassification of disease-infected seedlings as healthy (false negatives) poses the highest risk, as these plants may later fail or become unsellable. Conversely, misgrading healthy seedlings as defective (false positives) reduces contractor compensation and can lead to disputes. The current system performs well for aggregated quality grading, but disease and pest detection recall still falls below the ideal threshold required for large-scale industrial deployment.

## 5. Conclusions

This study develops an automated image-based quality assessment system that integrates deep learning, machine learning, and ensemble learning. The proposed three-stage grading method integrates top-view and multi-angle side-view imaging to detect leaf-surface defects, estimate root system conditions, and determine overall seedling quality grades. The hybrid grading framework, which combines YOLO-based deep learning detection with SVM and RF classification, enables the accurate and interpretable quality assessment of Phalaenopsis seedlings using multi-view RGB-D imagery. The system integrates automated feature extraction and decision-level reasoning to achieve reliable, scalable, and objective grading for orchid production.

Experimental results demonstrate that the three-stage grading method effectively identifies various types of leaf-surface defects, estimates root quantities, and classifies whole seedling quality with an *Overall_F1-score* of 84.44%, while the direct grading method provides faster processing with comparable accuracy (up to 90.44%) but lacks interpretability, as it cannot explain the reasoning behind its classification. The combination of these two approaches offers a practical balance, allowing rapid initial grading through the direct method, followed by detailed verification using the three-stage system for ambiguous samples.

Future research will focus on enhancing the robustness and scalability of the proposed grading system. Integrating hyperspectral or multispectral imaging could further improve defect discrimination beyond the visible spectrum, while real-time processing through embedded AI hardware or edge computing platforms would enable on-site grading in greenhouse or factory environments. Furthermore, developing a mobile-based interface could make the system more accessible for field or commercial applications. Expanding the dataset to include different orchid varieties, growth stages, and environmental conditions will further strengthen model generalization and adaptability to diverse nursery production scenarios.

## Figures and Tables

**Table 1:** Quantitative quality characteristic indicators for IQC of large-flowered 2.5-inch Phalaenopsis seedlings.

Defect Types	A Grade	B Grade	C Grade
(1) Diseases (a. Anthracnose, b. Yellow leaf disease, c. Phytophthora disease, d. Southern blight, e. Bacterial soft rot, f. Unknown spots)	None	b. Fine black lines appear on the stem.	b.: Slight yellowing spreads outward starting from the black lines on the stem. d.: White mycelium appears on the stem. c., e.: The leaf surface rapidly decays with a water-soaked appearance; in severe cases, fungal slime may develop. a., e., f.: Black circular spots with slightly yellow margins. a., c., f.: Dark green circular spots, light brown speckles, or irregularly shaped lesions appear on the leaf surface. a., c., f.: Circular spots appear on the leaf surface.
(2) Pest damage (Insect bite)	None	Circular or irregular bite marks appear on the lower leaves, regardless of size.	Circular or irregular bite marks appear on the upper leaves, regardless of size.
(3) Phytotoxicity (Pesticide damage)	None	Area of abnormal coloration on the upper leaf surface is <10–15% of the entire leaf area; Symmetrical deformation of the leaf outline affects <10–15% of the entire leaf area.	Area of abnormal coloration on the upper leaf surface is ≥10–15% of the entire leaf area; Symmetrical deformation of the leaf outline affects ≥10–15% of the entire leaf contour; Twisting and deformation of the leaf outline.
(4) Leaf damage	Length of midrib damage on the surface of the lower leaves is <1.5 cm.	Length of damage along the contour of a single leaf is <1.5 cm; Length of midrib damage on the surface of the upper leaves is <1.5 cm.	Length of damage along the contour of a single leaf is ≥1.5 cm; Length of midrib damage on the surface of the upper leaves is ≥1.5 cm.
(5) Leaf shrinkage	None	Upper leaves are shorter than the lower leaves, regardless of size.	None
(6) Leaf variation	None	Embossed patterns (leaf surface protrusions or depressions) or linear markings appear on the leaf surface, regardless of leaf size.	Leaf surface becomes twisted and deformed.
(7) Lower-leaf yellowing	None	Yellowing appears at the tips of the lower leaf surface, regardless of leaf size.	None
(8) Root system conditions	Root system ≥ 70%	50% < Root system < 70%	Root system ≤ 50%

**Table 2:** Root System Grading Standards.

Root Grade 3	Root Grade 2	Root Grade 1
≥3 roots and visual in =8 views	≥3 roots and visual in =7 views 0–1 root and visual in =1 view	≥3 roots and visual in ≤6 views 0–1 root and visual in ≥2 views
≥3 roots and visual in =7 views 2 roots and visual in =1 view	≥3 roots and visual in =6 views 2 roots and visual in =1 view 0–1 root and visual in =1 view	≥3 roots and visual in =4 views 2 roots and visual in =2 views 0–1 root and visual in =2 views
	≥3 roots and visual in ≤6 views 2 roots and visual in ≥2 views 0–1 root and visual in =0 views	≥3 roots and visual in ≤4 views 2 roots and visual in ≥2 views 0–1 root and visual in ≥2 views

**Table 3:** Performance indicator notation definitions for the classification and grading models in this study.

i	The *i*th category (defect type, root grade, or quality grade)
${TP}_{i}$	Number of samples in category *i* correctly classified as category *i*
${FP}_{i}$	Number of samples from other categories incorrectly classified as category *i*
${FN}_{i}$	Number of samples in category *i* incorrectly classified as other categories
$n_{i}$	Total number of samples in category *i*

**Table 4:** Performance indicator notation definitions for the regression prediction models in this study.

j	The *j*th category (regression type)
$y_{j}$	Actual value of the *j*th category
${\hat{y}}_{j}$	Predicted value of the *j*th category by the model
${\bar{y}}_{j}$	Mean value of the *j*th category
n	Number of samples

**Table 5:** Performance indicators of the optimal model combination at each stage of the three-stage grading method.

Stage 1			Stage 2		Stage 3
Top-view leaf defect detection	Side-view leaf defect detection	Side-view root count estimation	Side-view leaf defect count estimation	Side-view root system grading	Whole-seedling quality grading
YOLOv8	YOLOv10	YOLOv8	SVM-1	RF	SVM-2
73.20%	63.70%	92.40%	0.7026	89.43%	80.43%

**Table 6:** Performance indicators of category-wise classification for the single side-view leaf defect detection model in stage 1.

Category	*Precision*	*Recall*	*F1-Score*
Flawless	64%	86%	73%
Disease	87%	44%	58%
Pest damage	63%	20%	30%
Pesticide damage	87%	50%	64%
Leaf damage	79%	63%	70%
Leaf shrinkage	81%	66%	72%
Variation	84%	70%	77%
Lower-leaf yellowing	80%	77%	78%

**Table 7:** Confusion matrix of the three-stage grading method (all YOLOv8 models with 8 view-angles).

Actual\Predicted	A	B	C
A	15	4	2
B	7	84	5
C	3	9	24

**Table 8:** Confusion matrix of the direct grading method (Mix YOLOv8 and YOLOv10 models with 8 view-angles).

Actual\Predicted	A	B	C
A	17	1	3
B	4	89	3
C	4	2	30

**Table 9:** Per-class performance metrics for seedling quality grading by the three-stage grading method and the direct grading method.

Method	Class	*Precision*	*Recall*	*F1-Score*
Three-Stage	A	0.6000	0.7143	0.6512
	B	0.8660	0.8750	0.8705
	C	0.7742	0.6667	0.7164
	*Overall_F1 score*	—	—	0.8043
Direct Method	A	0.6800	0.8095	0.7380
	B	0.9674	0.9263	0.9464
	C	0.8333	0.8333	0.8333
	*Overall_F1 score*	—	—	0.8916

**Table 10:** Four viewing angle combinations based on selecting four images from the eight side views.

Viewing Angle Combination	0 Degrees	45 Degrees	90 Degrees	135 Degrees	180 Degrees	225 Degrees	270 Degrees	315 Degrees
4-1	✓	-	✓	-	✓	-	✓	-
4-2	-	✓	-	✓	-	✓	-	✓
4-3	✓	✓	-	✓	-	-	✓	-
4-4	-	-	✓	-	✓	✓	-	✓

**Table 11:** Four viewing angle combinations of selecting six images from the eight side views.

Viewing Angle Combination	0 Degrees	45 Degrees	90 Degrees	135 Degrees	180 Degrees	225 Degrees	270 Degrees	315 Degrees
6-1	✓	✓	✓	✓	-	✓	✓	-
6-2	✓	✓	-	✓	✓	-	✓	✓
6-3	✓	✓	✓	✓	-	-	✓	✓
6-4	✓	✓	-	✓	✓	✓	✓	-

**Table 12:** Four viewing angle combinations of selecting six and five images from the eight side views.

Viewing Angle Combination	0 Degrees	45 Degrees	90 Degrees	135 Degrees	180 Degrees	225 Degrees	270 Degrees	315 Degrees
6-5	✓	-	-	✓	✓	✓	✓	✓
6-6	✓	-	✓	✓	✓	-	✓	✓
5-1	✓	-	-	✓	✓	-	✓	✓
5-2	✓	-	-	✓	✓	✓	-	✓

**Table 13:** Six viewing angle combinations of selecting four images from the eight side views.

Viewing Angle Combination	0 Degrees	45 Degrees	90 Degrees	135 Degrees	180 Degrees	225 Degrees	270 Degrees	315 Degrees
4-1	✓	-	✓	-	✓	-	✓	-
4-5	✓	-	✓	✓	-	-	✓	-
4-6	✓	-	✓	-	✓	-	-	✓
4-7	✓	-	-	✓	✓	-	✓	-
4-8	✓	-	-	✓	✓	-	-	✓
4-9	✓	-	-	✓	-	-	✓	✓

**Table 14:** Two viewing angle combinations of selecting five images from the eight side views.

Viewing Angle Combination	0 Degrees	45 Degrees	90 Degrees	135 Degrees	180 Degrees	225 Degrees	270 Degrees	315 Degrees
5-2	✓	-	-	✓	✓	✓	-	✓
5-3	✓	-	✓	✓	-	-	✓	✓

**Table 15:** Optimal viewing angle combinations corresponding to different numbers of deleted side-view angles.

Number of Deleted Side-View Angles	1	2	3	4
Viewing angle combination	7-2	6-6	5-3	4-5
*F1-score*	81.12%	82.50%	81.22%	79.72%

**Table 16:** Performance comparison of different YOLO model combinations applied in stage 1 of the three-stage grading approach.

Performance Indices	All in YOLOv8	All in YOLOv10	YOLOv8 + YOLOv10
*Overall_F1-score* (%)	82.30%	79.80%	80.43%
Total training time (Min.)	394.02	407.92	436.18
Testing time/seedling (s)	1.9284	1.9752	1.9784

**Table 17:** Performance comparison of different YOLO model combinations applied in the direct grading approach.

Performance Indices	All in YOLOv8	All in YOLOv10	YOLOv8 + YOLOv10	YOLOv10 + YOLOv8
*Overall_F1-score* (%)	73.86%	89.79%	89.79%	73.29%
Total training time (Min.)	239.48	295.07	276.26	299.44
Testing time/seedling (s)	1.2181	0.8272	0.8364	1.2442

## Data Availability

The original contributions presented in this study are included in the article/Supplementary Material. Further inquiries can be directed to the corresponding author.

## Supplementary Materials

The following supporting information can be downloaded at: https://www.mdpi.com/article/10.3390/s25247502/s1, Figure S1: Images of the six main diseases of Phalaenopsis potted seedlings; Figure S2: Images of three types of pest damage affecting Phalaenopsis potted seedlings; Figure S3: Three common images of pesticide damage to Phalaenopsis potted seedlings; Figure S4: Images of leaf morphology disorders of Phalaenopsis potted seedlings; Figure S5: Images of leaf surface issues in Phalaenopsis potted seedlings; Figure S6: Images of three root conditions of Phalaenopsis potted seedlings; Figure S7: User interface of the classification and grading system developed in this study; Table S1: Percentage distribution of the seven leaf-defect categories in the dataset; Table S2: Percentage distribution of root-system levels in the dataset; Table S3: Percentage distribution of seedling quality grades in the dataset; Table S4: Number of seedling samples required for each model in different experimental stages; Table S5: Parameter settings of deep learning YOLO models; Table S6: Parameter settings of machine learning SVM and RF models; Table S7: Confusion matrix of category-wise classification for the single side-view leaf defect detection model in stage 1; Example S1: An example of transforming detection outputs into structured feature vectors for SVM seedling grading.
