# Supplementary material for: Smart Image-Based Deep Learning System for Automated Quality Grading of Phalaenopsis Seedlings in Outsourced Production
Source: Sensors (Basel). 2025 Dec 10;25(24):7502. doi: 10.3390/s25247502 (PMC12737025; doi:10.3390/s25247502)
Supplement: Supplementary file 1 [file sensors-25-07502-s001.zip › sensors-3992928-supplementary.pdf]

Supplementary Material

Supplementary Figure S1:

Six major diseases are observed in Phalaenopsis seedlings.

Diseases (Six major diseases)

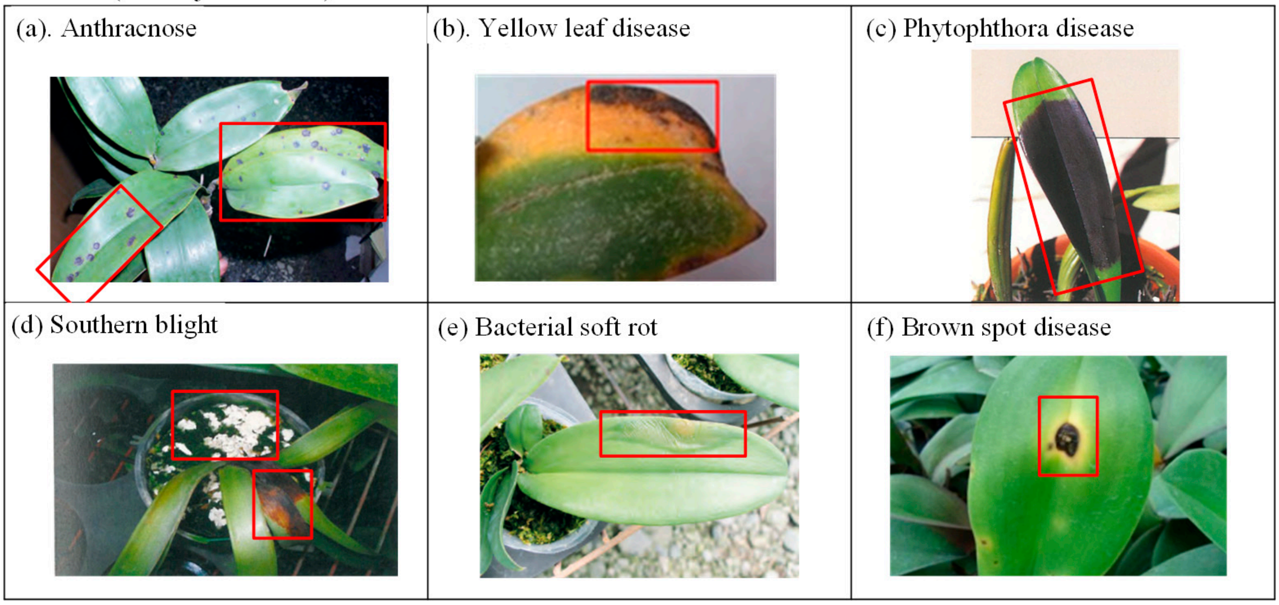

Supplementary Figure S1. Images of the six main diseases of Phalaenopsis potted seedlings.

Supplementary Figure S2:

Three major insect damages in Phalaenopsis seedlings.

Pest damage (Three major pests: Aphid\ Scale insects\ Thrips)

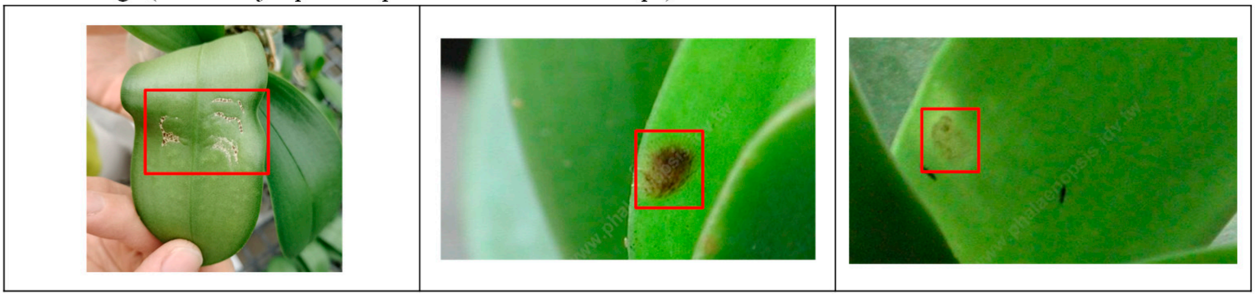

Supplementary Figure S2. Images of three types of pest damage affecting Phalaenopsis potted seedlings.

Supplementary Figure S3:

Three common types of chemical damage in Phalaenopsis seedlings.

Pesticide damage (Three common types of phytotoxicity: Vinclozolin\ Dichlofluanid\ Metiram-vinclozolin)

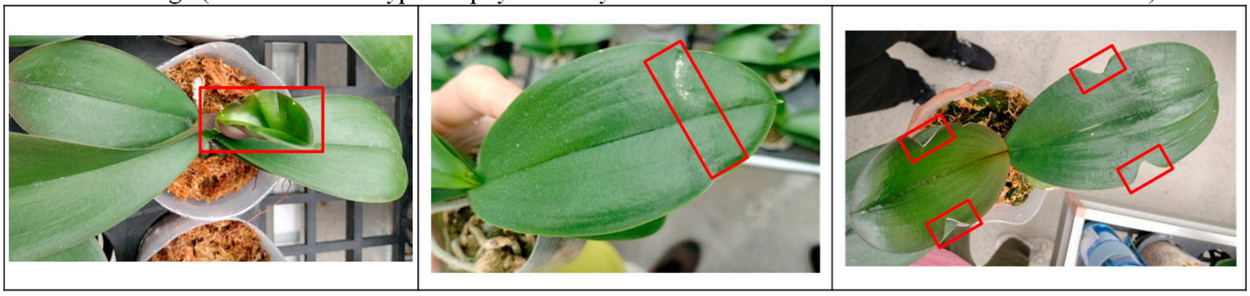

Supplementary Figure S3. Three common images of pesticide damage to Phalaenopsis potted seedlings.

Supplementary Figure S4:

Examples of leaf damage and shrinkage in Phalaenopsis seedlings.

Leaf morphology disorders (Leaf damage/ Leaf shrinkage)

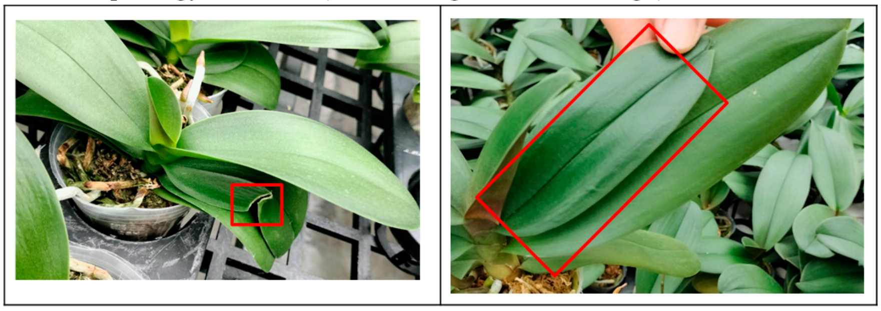

Supplementary Figure S4. Images of leaf morphology disorders of Phalaenopsis potted seedlings.

Supplementary Figure S5:

Examples of leaf variation and lower-leaf yellowing in Phalaenopsis seedlings.

Leaf surface issues (Leaf variation/ Yellowing of lower leaves)

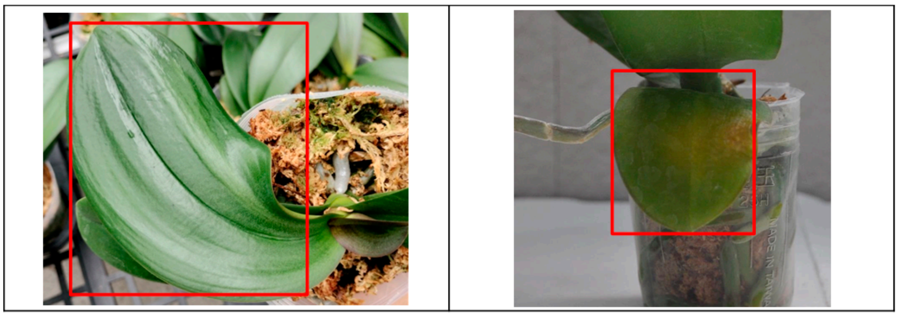

Supplementary Figure S5. Images of leaf surface issues in Phalaenopsis potted seedlings.

Supplementary Figure S6:

Three root conditions in Phalaenopsis seedlings.

Root system issues

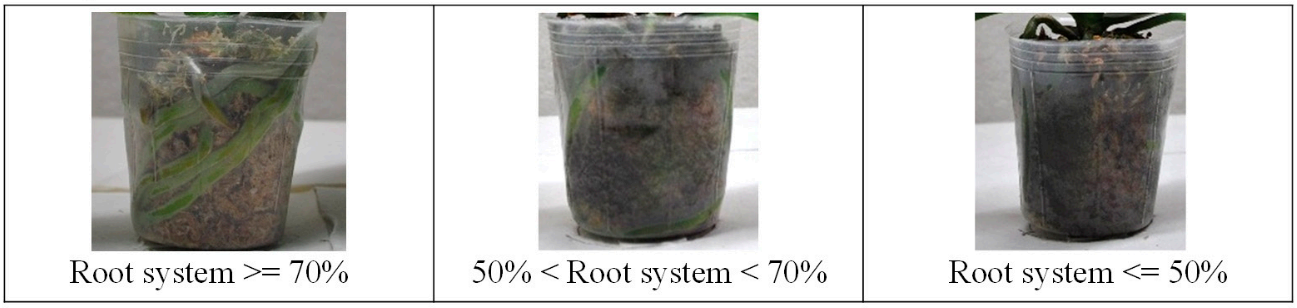

Supplementary Figure S6. Images of three root conditions of Phalaenopsis potted seedlings.

Supplementary Figure S7:

Figure presents the user interface of the automated classification and grading system for outsourced Phalaenopsis potted seedlings developed in this study. The interface integrates top-view RGB-D and multi-angle side-view images for defect detection, object estimation, and quality grading.

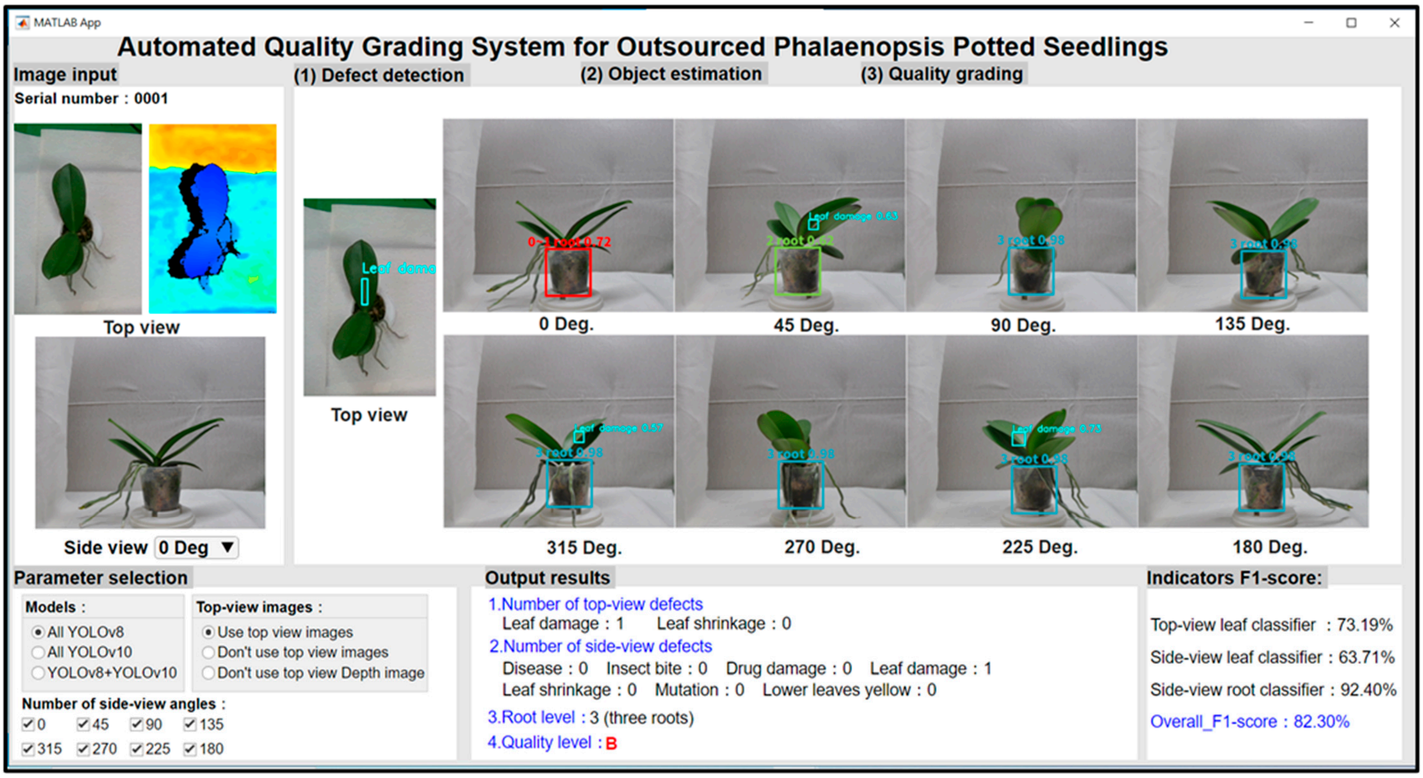

Supplementary Figure S7. User interface of the classification and grading system developed in this study.

Supplementary Tables S1-S3.

The complete distribution of defect categories, root system levels, and seedling quality grades used in this study is summarized in the supplementary tables below.

Supplementary Table S1. Percentage distribution of the seven leaf-defect categories in the dataset.

Category	Diseases	Pest damage	Pesticide damage	Leaf damage	Leaf shrinkage	Leaf variation	Lower-leaf yellowing
Percentage	16%	15%	13%	18%	2%	12%	24%

Supplementary Table S2. Percentage distribution of root-system levels in the dataset.

Root system levels	Level-3	Level-2	Level-1
Percentage	87%	11%	2%

Supplementary Table S3. Percentage distribution of seedling quality grades in the dataset.

Seedling quality grades	Grade-A	Grade-B	Grade-C
Percentage	22%	55%	23%

Supplementary Table S4.

Dataset composition for quality grading of Phalaenopsis potted seedlings, showing the number of training and testing images across all categories.

Supplementary Table S4. Number of seedling samples required for each model in different experimental stages.

(1) Parameter settings for small-sample experiments (B/C defective seedlings)			
	Training	Validation	Testing
Top-view leaf defect detection	40	10	10
Side-view leaf defect detection	209	68	67
Side-view root count estimation	210	70	61
Side-view leaf defect count estimation	210	70	70
Side-view root system grading	180	60	60
Whole-seedling quality grading	180	60	60
(2) Model selection in large-sample experiments (B/C defective seedlings)			
	Training	Validation	Testing
Top-view leaf defect detection	100	15	35
Side-view leaf defect detection	596	76	67
Side-view root count estimation	559	97	94
Side-view leaf defect count estimation	465	117	103
Side-view root system grading	720	240	240
Whole-seedling quality grading	618	132	133
(3) Overall system performance comparison (Test set: A/B/C seedlings)			
Test sample size			153
(4) Sensitivity analysis (Test set: A/B/C seedlings)			
Test sample size			153

Supplementary Table S5.

This study evaluates the YOLO models using the Overall F1-score to identify the optimal combination of parameter settings that yielded the best detection performance.

Supplementary Table S5. Parameter settings of deep learning YOLO models.

Module	Optimizer	Learning rate	Batch size
Top-view leaf defect detection	Adam	0.001	8
Side-view leaf defect detection	SGD	0.001	8
Side-view root count estimation	SGD	0.001	12

Supplementary Table S6.

This study evaluates the SVM and RF models using the R^2 score and Overall F1-score to identify the optimal combination of parameter settings that yielded the best detection performance.

Supplementary Table S6. Parameter settings of machine learning SVM and RF models.

Module	Parameter-1	Parameter-2	Parameter-3
Estimation of leaf-surface defect counts (SVM-1)	Kernel function: Linear	Penalty parameter: C=0.1	Epsilon-insensitive loss function: ε=0.01
Root system grade of potted seedlings (RF)	Number of decision trees in the forest: 200	Maximum number of depths in each decision tree: 15	Minimum number of samples required to split an internal node: 2
Quality grading of whole potted seedlings (SVM-2)	Kernel function: Linear	Penalty parameter: C=1	kernel coefficient: γ=scale

Supplementary Table S7.

To further analyze the misclassification patterns of the single side-view leaf-defect detection model in stage 1, a category-wise confusion matrix is generated, summarizing how each leaf defect type is predicted.

Supplementary Table S7. Confusion matrix of category-wise classification for the single side-view leaf defect detection model in stage 1.

Category	Flawless	Disease	Insect damage	Pesticide damage	Leaf damage	Leaf shrinkage	Variation	Lower-leaf yellowing	Total
Flawless	653	9	15	7	22	5	11	41	763
Disease	75	59	-	-	-	-	-	-	134
Pest damage	107	-	26	-	-	-	-	-	133
Pesticide damage	47	-	-	46	1	-	-	-	94
Leaf damage	50	-	-	-	85	-	-	-	135
Leaf shrinkage	11	-	-	-	-	21	-	-	32
Variation	25	-	-	-	-	-	59	-	84
Lower-leaf yellowing	49	-	-	-	-	-	-	162	211
									1586

Supplementary Example S1. An example of transforming detection outputs into structured feature vectors for SVM seedling grading.

A concrete example of how raw defect-detection outputs and root-count predictions are converted into the structured feature vector used by the SVM classifier for whole-seedling quality grading. Each YOLO detection provides a tuple *(class, x_center, y_center, width, height)* normalized to [0, 1]. During transformation, detections belonging to the same defect category across eight side-view images are aggregated to compute: (1) the total number of occurrences, (2) the weighted defect area, and (3) the presence/absence indicator (binary flag). For example, if the “leaf damage” class (class ID = 3) is detected three times across eight views with areas 0.012, 0.009, and 0.015, the transformed features become:

- Count_feature_damage = 3
- Weighted_area_damage = 0.012 + 0.009 + 0.015 = 0.036
- Presence_flag_damage = 1

This transformation converts variable-length detection outputs into a fixed-length, 17-dimensional feature vector that can be consistently used for quality grading.

Example of Data Transformation for Feature Vector Construction

1. **Raw Inputs From Detection Models**
   - **Side-view leaf defect detections (8 views):**
     - View-0: Disease(1), Leaf damage(0), Pest(1), Shrinkage(0), Variation(0), Lower-leaf yellowing(0), Pesticide(0)
     - View-45: Disease(0), Leaf damage(1), Pest(0), Shrinkage(0), Variation(0), Lower-leaf yellowing(0), Pesticide(0)
     - … (remaining six views)
   - **Side-view root counts (8 views):**
     - [3, 2, 3, 3, 2, 3, 3, 3]
   - **Top-view RGB-D defect presence:**
     - Leaf damage: present (1)
     - Leaf shrinkage: absent (0)
2. **Transformation into Aggregated Defect Features**
   - **Per-class total counts (7 classes):**
     - Disease = 2
     - Leaf damage = 3
     - Pest = 1
     - Shrinkage = 0
     - Variation = 0
     - Lower-leaf yellowing = 0
     - Pesticide = 0
   - **Weighted defect areas:**
     - Disease = 112.4
     - Leaf damage = 98.7
     - Pest = 36.2
     - Remaining classes = 0

3. **Transformation into Root Features**
   - **Root grade from multi-view rule:**
     - View count of ≥3 roots = 6
     - Assigned root grade = Grade 3
4. **Final Structured Feature Vector (17 Dimensions)**

$$\begin{aligned}\mathbf{F} = [&\text{LeafDamage}_{top} = 1,\ \text{LeafShrinkage}_{top} = 0,\\ &\text{DefectTotal}_{7\ classes} = (2, 3, 1, 0, 0, 0, 0),\\ &\text{WeightedArea}_{7\ classes} = (112.4, 98.7, 36.2, 0, 0, 0, 0),\\ &\text{RootGrade} = 3]\end{aligned}$$

This feature vector is then standardized and used as input to the SVM classifier.
